# Systemic Glycosaminoglycan Clearance by HARE/Stabilin-2 Activates Intracellular Signaling

**DOI:** 10.3390/cells9112366

**Published:** 2020-10-28

**Authors:** Paul H. Weigel

**Affiliations:** Department of Biochemistry and Molecular Biology, University of Oklahoma Health Sciences Center, Oklahoma City, OK 73104, USA; paul-weigel@ouhsc.edu; Tel.: +1-405-271-1288

**Keywords:** clathrin-coated pit, ERK1/2, phagocytosis, receptor isoform, receptor-mediated endocytosis, receptor recycling, scavenger receptor, systemic clearance

## Abstract

Scavenger receptors perform essential functions, critical to maintaining mammalian physiologic homeostasis by continuously clearing vast numbers of biomolecules from blood, interstitial fluid and lymph. Stabilin-2 (Stab2) and the Hyaluronic Acid Receptor for Endocytosis (HARE), a proteolytic isoform of Stab2, are important scavenger receptors responsible for the specific binding and internalization (leading to degradation) of 22 discrete molecules, macromolecular complexes and cell types. One-third of these ligands are glycosaminoglycans (GAGs). Full-length Stab2, but not HARE, mediates efficient phagocytosis of apoptotic cells and bacteria via binding to target surface ligands. HARE, the C-terminal half of Stab2, mediates endocytosis of all the known soluble ligands. HA was the first ligand identified, in 1981, prior to receptor purification or cloning. Seven other GAG ligands were subsequently identified: heparin, dermatan sulfate, chondroitin and chondroitin sulfates A, C, D and E. Synthetic dextran sulfate is also a GAG mimic and ligand. HARE signaling during HA endocytosis was first discovered in 2008, and we now know that activation of HARE/Stab2 signaling is stimulated by receptor-mediated endocytosis or phagocytosis of many, but not all, of its ligands. This review focuses on the HARE-mediated GAG activation of intracellular signaling, particularly the Extracellular Signal-Regulated Kinase 1/2 pathway.

## 1. Introduction

Scavenger receptors (SRs^1^). The name itself for this class of cell surface receptors may signify to some that their physiologic functions are not important or necessary. However, as those living throughout the world know well, if trash and abandoned items in their neighborhoods are not removed, their daily routines and living conditions can quickly deteriorate. Likewise, when a scavenging or “housekeeping” function is impaired or absent, the physiologic consequences may cause significant health impairment or disease [1]. The PrabhuDas et al. report [1] summarizes the recommendations of a working panel of 15 experts charged, by the US National Institutes of Health, with developing a comprehensive consensus definition and classification of scavenger receptors, as well as a standardized nomenclature. The panel’s recommendations were then presented for feedback and comments at three national meetings, ultimately resulting in the revised final 2017 publication. They officially defined SRs as functioning “… by mechanisms that include endocytosis, phagocytosis, adhesion, and signaling that ultimately lead to the elimination of degraded or harmful substances.” The final classification scheme proposed ten classes for the 28 known receptors. The SR-H2 classification includes Stab2 and HARE [1].

This overview focuses on the binding and endocytosis of GAGs mediated by HARE and Stab2, with emphasis on current knowledge about the subsequent activation of cell signaling. Although these two protein isoforms (Section 5) are expressed primarily in macrophages and sinusoidal endothelial cells (SECs), there are few if any studies investigating soluble GAG endocytosis by either Stab2 or HARE in circulating macrophages. In contrast, many groups have studied CD44-mediated macrophage phagocytosis of particles coated with hyaluronan (HA). Therefore, most of the current relevant knowledge for this article is based on the function of HARE, rather than Stab2, in SECs and recombinant human cell lines. It remains to be determined if full-length Stab2 similarly activates signaling in response to soluble GAG endocytosis, although this cannot currently be assessed until Stab2 can be expressed without the ubiquitous and concurrent generation of the HARE isoform (Section 5.3).

## 2. Glycosaminoglycans (GAGs)

**Figure 1:**
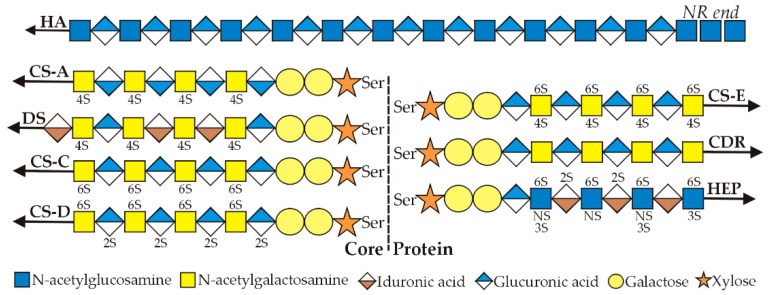
Glycosaminoglycan diversity. The schematic representation of glycosaminoglycans (GAG) structures shows that heparin (Hep), dermatan sulfate (DS), chondroitin (CDR), chondroitin sulfates A (CS-A), C (CS-C), D (CS-D), and E (CS-E), but not HA, are synthesized onto core proteins (dashed line), which can be membrane-bound or soluble, via a common linker sequence (Galactose-Galactose-Xylose; two circles-star) attached to Ser. All GAGs are made as linear polymers with repeating disaccharide units containing either N-acetylglucosamine (GlcNAc, blue square) or N-acetylgalactosamine (GalNAc, yellow square) and either glucuronic acid (GlcU, blue diamond) or iduronic acid (IdU, brown diamond). Arrows denote the polymer growing end, to which new sugars are added. Sulfation occurs during biosynthesis and different cell types can create different negative charge patterns (unique molecular signatures) that are specifically recognized by other soluble or membrane proteins (e.g., Hyaluronic Acid Receptor for Endocytosis (HARE)). GAGs built on a core protein are attached at their reducing end, and new sugars are added at the nonreducing end. In contrast, HA is not attached to or built onto a core protein. Rather, HA synthesis is initiated de novo and extended by HA synthase until the UDP-HA chain is released, at which point it cannot be rebound and extended further [2]. Additionally different is that HA synthesis occurs at the reducing end, which is still attached to UDP. Thus, the first sugars assembled remain at the nonreducing end (NR end) and the linkages in this unique region are chitin [GlcNAc(β1,4)_n_] rather than HA. HA synthase initiates HA disaccharide synthesis only after first assembling a chitin-UDP (UDP-GlcNAc_3-4_) primer for starting HA synthesis [2]. The primer then remains as a chitin cap at the NR end [3].

GAGs are virtually ubiquitous on cell surfaces, in fluids and in extracellular biomatrices throughout the body. HARE/Stab2 recognizes 8 of the 10 GAGs typically found in mammals (Figure 1); all but keratan sulfate and heparan sulfate. 

All GAGs are linear chains comprised of repeating disaccharide units, each containing a uronic acid (i.e., glucuronic or iduronic) and an N-acetylamino sugar (i.e., glucosamine or galactosamine). All GAGs except HA are synthesized by multiple enzymes via specific linkages to one of at least 28 different Core Proteins [4,5].

In sharp contrast, HA synthesis is initiated and extended by a single enzyme [2,3], HA synthase, first molecularly cloned in 1993 by the author’s group [6]. HA is also the only GAG that is not sulfated. All other GAGs are sulfated on one or both sugars of disaccharide units throughout the polymer, creating an enormous number of different sulfation patterns. Chondroitin (CDR) is not sulfated, whereas chondroitin sulfate A (CS-A), C (CS-C), D (CS-D) and E (CS-E) are each sulfated at different specific positions (Figure 1), creating different negative charge patterns within the polysaccharide. Nonetheless, these are all identical sugar polymers. What was formerly called CS-B was renamed as dermatan sulfate (DS), since it is a different sugar polymer backbone than all the CS GAGs (Figure 1).

Another significant difference between HA and all other GAGs is that HA can be ≥100-times longer, with a molecular mass as large as 10 MDa [7]. Other GAGs are typically much smaller, with molecular masses <50 kDa.

## 3. Glycosaminoglycan and Proteoglycan Turnover, Biomatrix Plasticity and Recycling Receptor-Mediated Endocytosis

### 3.1. Systemic GAG Clearance Is also a Mechanism for Turnover of Proteoglycans (PGs) and Other Bound (Piggybacking) Proteins

All the GAGs except HA are covalently attached to core proteins (Figure 1). As for any biomolecule, each of these soluble or cell surface PGs (in biomatrices and on tissue or circulating cells) turns over with a characteristic and specific half-life. Proteins that are not heavily glycosylated generally turnover via proteolytic cleavage processes. In contrast, many PGs are so heavily decorated with GAG chains and other N- or O-linked glycans that their core proteins are minimally accessible to proteases [5]. Their partial cleavage, therefore, releases large soluble PG fragments that are readily cleared from the blood and lymphatic circulatory systems by GAG-specific rather than protein-specific receptors.

Decades before the molecular cloning and identification of HARE and Stab2, this PG clearance function was demonstrated by Eskild et al. [8], who found that CS-PGs injected into rats were rapidly cleared from the blood by the liver HA clearance receptor (aka HARE). Similarly, any other proteins that are bound to GAG chains or to the PG core protein will also be bound and endocytosed, indirectly by HARE, as part of the large GAG–PG complex. For example, the large (~2 MDa) cartilage PG aggrecan assembles into macromolecular structures containing up to 50 PGs bound to a single HA chain; an extremely large complex [5].

Thus, many different types of proteins are cleared and have at least part of their turnover controlled by continuous GAG-recognition and uptake processes that result in molecular degradation in lysosomes.

### 3.2. Morning Stiffness, Flexibility and Biomatrix Plasticity

An under-recognized purpose of the normal turnover of multiple components, particularly HA, within biomatrices throughout the body is to avoid the deleterious consequences if this process slows or is inhibited: matrix crosslinking leading to rigidification and loss of tissue plasticity. In short, in the absence of ongoing regular (e.g., daily) turnover of individual biomatrix components, any tissue would continually accumulate new covalent bonds among all its molecular components. Free radical or oxidative crosslinking, O-esterification and trans-N-acyl reactions likely occur more frequently in tissues, compared to lab conditions, due to the very high reactant concentrations within tissues.

Our tissues would, essentially, turn into stiffer and stiffer gels until tissue function, as well as physical movement, were impaired enough that viability was at risk. Decreased biomatrix pliability would also impede the movement of infiltrating macrophages and other immune cells that regularly monitor tissues and remove foreign pathogens and debris. The tissue plasticity concept may sound dramatic but if you experience morning stiffness, this is part of the reason [9]. Therefore, it makes bio-sense that mechanisms to achieve controlled and continuous biomatrix turnover have evolved to minimize the risk of losing normal tissue flexibility, pliability and function.

### 3.3. The Importance of Constitutive Receptor-Mediated Endocytosis and Receptor Recycling

The GAG trash burden and very large mass of biomolecules removed from body tissues daily, noted in Section 3.1, require a very active and efficient system for their binding, internalization and degradation. Unlike many receptors that are only internalized after they bind to a specific ligand, the HARE and Stab2 clearance receptors are continuously recycling receptors that move to and bind clathrin in forming coated pits as part of a constitutive coated pit endocytosis pathway [10].

This constitutive endocytosis pathway results in the ongoing targeting to coated pits and uptake of free receptors or receptor–ligand complexes, dissociation of bound ligands from receptors, recycling of receptors back to the cell surface and delivery of the released free ligands to lysosomes for degradation. The process can be visualized as a continuously moving or airport baggage claim belt [10], on which the luggage (receptor) constantly moves from the cell surface to multiple cell interior compartments and back to the surface with or without contents (ligand). Requiring no activation, recycling receptors are always working—moving between the surface and interior, 24/7.

### 3.4. The GAG and GAG-PG “Trash Problem”

HA and all the CS-PGs are components of extracellular biomatrices in all vertebrate tissues and they are continuously synthesized and degraded in these tissues throughout the body. Their continuous cleavage and removal from tissues, followed by their subsequent uptake (e.g., by HA-specific receptors) into the vascular and lymphatic circulatory networks and subsequent destruction by specialized SECs, is important for maintaining physiologic health and homeostasis [11].

To understand the importance of the GAG turnover system, we can estimate the extent of the normal daily GAG breakdown in our total body tissues. For example, HA is partially cleaved and large fragments are continuously released from biomatrices throughout the body. The total body HA content of a 70 kg person is about 15 g [12] and the amount of HA turned over daily (degraded and replaced by new biosynthesis) is up to 5 g.

Thus, the overall turnover rate for whole body HA is ~3 days; as expected, some HA pools turnover much slower and some faster [13]. In the case of the dermis, our largest organ with ~50% of total body HA, the HA initially deposited as large MDa HA has a degradation half-life of ~24 h [14].

If the 5 g of daily HA “trash” was not removed (remaining in our blood, lymph and other interstitial fluids), then by age 2 we would accumulate ~3.7 kg (8 lb) of HA fragments! Developing multiple pathologies would be inevitable. Similarly, there is also a continuous new synthesis of the many other PG proteins and their associated GAGs and their subsequent turnover. A large fraction of released GAG chains is still attached to core protein fragments that were cleaved as part of their normal turnover process [4,5]. Thus, the total mass of GAGs, PG-GAG fragments and piggybacking proteins released from biomatrices is much more than that for HA alone, likely >5 g/day.

### 3.5. High Capacity GAG Uptake and Degradation Is Mediated by Stab2/HARE in SECs

To understand the importance of SRs such as HARE/Stab2, we need to consider the consequences for our health and functionality of not “removing the trash”. A typical receptor recycling round-trip is ~7 min, so each HARE receptor traverses ~200 cycles/day delivering ligands to lysosomes for degradation [10]. Since the liver, spleen, lymph node or bone marrow SECs have about 500,000 HARE/Stab2 receptors per cell [15], each SEC can internalize and degrade 10^8^ GAGs or GAG-PGs each day.

In the human liver, the number of SECs (40% the number of hepatocytes) is about 5 × 10^6^/g, so a typical 1.4 kg liver has about 7 × 10^9^ SECs. Thus, Stab2/HARE-mediated clearance by liver SECs is estimated at 7 × 10^17^ GAG-containing molecules daily. The total SEC number in the human spleen, lymph nodes and other tissues expressing Stab2/HARE [16,17] likely increases the total body HARE/Stab2 turnover capacity to >10^18^ GAG-ligands/day!

As explained in Section 1 and Section 5, there are no published data showing that full-length Stab2 mediates GAG or other ligand endocytosis as effectively as the half-length Stab2 isoform HARE. This is because both isoforms occur in all isolated SECs or recombinant cells expressing full-length Stab2, which is ubiquitously proteolyzed to make HARE. To date, expression of HARE alone (with Stab2 absent) has only been achieved in transfected cells expressing a recombinant HARE gene.

## 4. Full-Length Stab2 Mediates Macrophage Binding to and Engulfment of Apoptotic Cells and Bacteria

**Figure 2:**
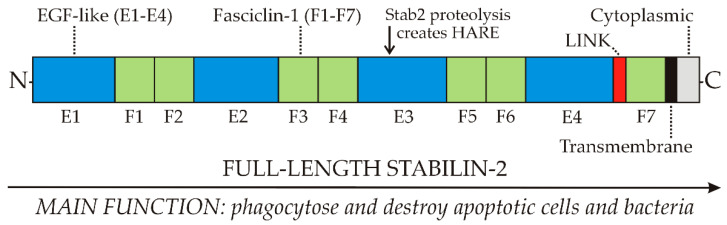
Human Stab2 domain organization. Full-length Stab2 is a type I membrane protein [17] of 2551 aa with a large N-terminal extracellular domain, a 92 aa HA-binding Link module (red), a 22 aa transmembrane domain (TMD, black), and a 72 aa C-terminal cytoplasmic domain (CD, gray). The ectodomain contains 7 Fasciclin-1 domains (F1–F7; green) and 4 larger EGF-like domains (blue; E1–E4) spanning the ectodomain length. Multiple binding sites for phosphatidylserine (PS) and for αMβ2 and αvβ5 integrins are within the E1-E4 and F1–F7 domains, respectively. PS binding sites enable receptor recognition of dying apoptotic cells [18] and integrin-binding sites enable receptor recognition of lymphocytes [19] in coordination with PS interactions [20]. A proteolytic cleavage site (arrow) that generates the 190 kDa HARE isoform at Ser^1136^ [21] is within Fasciclin-1 domain 4 (F4). This constitutive cleavage process gives two half-receptors: a soluble N-terminal half, whose fate is unknown, and the membrane-bound C-terminal half that is HARE (Section 5).

Stab2 on macrophages binds and enables phagocytoses of apoptotic red blood cells, apoptotic lymphocytes and both Gram-negative and Gram-positive *bacteria* (Figure 2). 

Kim, Park and colleagues have extensively and elegantly characterized the receptor domains involved in the recognition of phosphatidylserine (PS) and integrins [18,19,20,22]. Three cell-associated molecules that are ligands for Stab2-mediated engulfment have been identified: PS, αMβ2 integrin and αvβ5 integrin. PS binding sites are within the four Epidermal Growth Factor (EGF)-like domains E1-E4; integrin-binding sites are within the seven Fasciclin domains, F1–F7.

Thus, full-length Stab2 has multiple domains throughout that bind to PS and integrins on cell surface membranes (Figure 2). Stab2 does not bind to normal healthy cells because they have little PS in their outer membrane leaflets. In contrast, apoptotic cells in the process of dying are less able to control this asymmetric PS distribution and more PS moves to the cell surface. Macrophages expressing Stab2 are then able to recognize, bind, phagocytose and destroy daily large numbers of apoptotic cells, such as dying red or white blood cells.

## 5. The HARE Isoform Is Created In Vivo and In Vitro by Controlled Partial Stab2 Proteolysis

### 5.1. HARE Is a Stab2 Protein Isoform, Not a Splice Variant

It is critical to note that HARE is not a splice variant of Stab2. Although Hare and Harris [23] found multiple Stab2 splice variants in the human liver, spleen and lymph nodes, the HARE protein is not created as a splice variant. No HARE mRNA encoding HARE was found. Significantly, human HARE is produced by proteolysis of full-length recombinant Stab2 [21]. Using a full-length cDNA coding sequence, mRNA splicing is not possible since no introns are present in a recombinant gene.

Nonetheless, 2551 aa recombinant full-length human Stab2 is cleaved uniquely at the N-terminal Ser^1136^ bond to create the 1416 aa HARE isoform, a 190 kDa type 1 membrane receptor with a core protein predicted mass of 154,090 Da and a pI of pH 5.9 [24]. HARE is designated here as the C-terminal half of Stab2.

**Figure 3:**
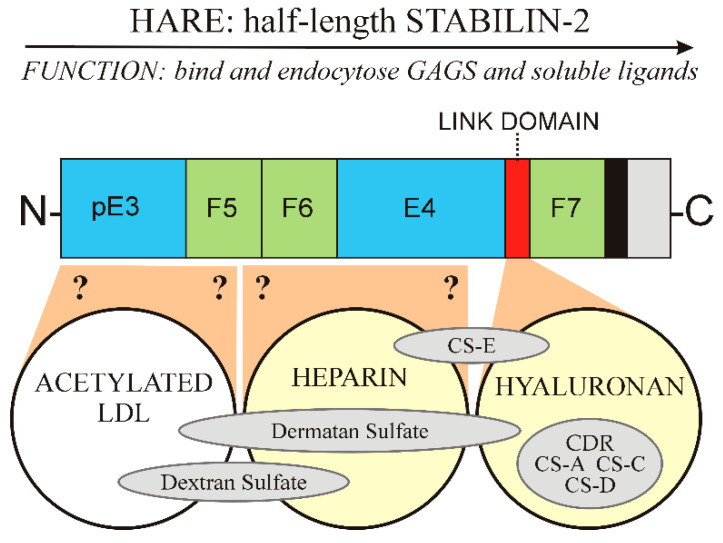
All GAG and other soluble ligand-binding domains are within the HARE portion of Stab2. The half-receptor HARE isoform (1416 aa) is the C-terminal half of Stab2 and contains the CD (gray), membrane (black) and Link (red) domains, as well as Fasciclin-1 domains F5–F57 and EGF-like domain E4 of the full-length protein (Figure 2). The N-terminal region of HARE is a truncated portion of the Stab2 E3 domain (pE3). All known GAG binding sites (yellow circles and gray ellipses) are within HARE [24,25]. The Link module enables binding to HA, CDR, CS-A, CS-C and CS-D (right yellow circle); these four CDR GAGs inhibit HA binding and Link module deletion eliminates all five binding activities [26]. A separate Hep binding site is on the N-terminal side of the Link domain. A third independent binding site for acetylated low-density lipoprotein (AcLDL; white circle), is assigned to the N-terminal side of the Hep site. Based on competition studies [26], DS binds to or occludes the Hep binding site and partially blocks both HA and AcLDL binding. DxS competes with both Hep and AcLDL binding but does not block HA binding. CS-E competes at a low level with both HA and Hep but does not affect AcLDL binding.

The 1416 aa HARE isoform (Figure 3) is 55.5% of full-length Stab2 and a fully functional coated pit targeted recycling receptor [25,26]. If Stab2 processing occurs intracellularly or at the cell surface is unknown. The fate of the released soluble N-terminal receptor half is also not known. It is also intriguing to consider that the N-terminal half-Stab2 isoform could be functional after cleavage as a soluble circulating protein.

### 5.2. Primary and Recombinant Cells Express Distinct 2:1 Ratios of HARE:Stab2

SECs in the liver, spleen and lymph nodes are the major tissues responsible for circulating GAG clearance, and these cells express the same amounts of HARE and Stab2 protein. Consistent with HARE having roughly half the aa number as Stab2, immuno-purification yields, Western blot assays and HA-binding ligand blot assays all show that liver, lymph node and spleen SECs express twice as much HARE as Stab2, on a molar basis [24,25,26,27,28,29]. Therefore, to maintain a 2:1 molar ratio of the 190:315 kDa Stab2 isoforms, the SECs in these tissues must process and cleave only two-thirds of the continuously synthesized Stab2 proteins. Achieving the observed net molar ratio (2:1) of two half-length HARE proteins for each full-length Stab2 protein, readily occurs if only two out of every three Stab2 proteins synthesized are proteolytically cleaved to produce two HARE. Since one out of three Stab2 proteins remain intact, the ratio of the two species is set at 2:1 (HARE:Stab2). It is challenging to envision how cells can control this Stab2 cleavage process, so it is only partial rather than complete cleavage, as typically occurs.

Every cell line that we created, proteolytically cleaves the same fraction of the Stab2 made to create a mixture of both HARE and Stab2 [21]. Consequently, this situation of dual-functional receptors has complicated studies to assess the ligand binding capability of full-length Stab2 alone.

### 5.3. All Stab2 Studies Unknowingly Include Data Reflecting the Ubiquitous HARE Isoform

All in vitro cell-based experiments reflect the presence and function of two different receptor populations, because the inherent intracellular proteolytic processing of full-length Stab2 creates the two functional receptor isoforms. Politz et al. [17] and Prevo et al. [30] found that Stab1 (aka, FEEL-1 and CLEVER-1) is processed in a comparable way to produce two protein isoforms of similar size to the Stab2 isoforms.

Prevo et al. [30] also showed that the Stab1 Link module does not bind HA, a significant functional difference between Stab1 and Stab2. Circulating macrophages may express more Stab2 than HARE [17], but this has not yet been examined in detail by groups focused only on Stab2 and not also recognizing the presence of HARE; some reports do not include relevant data for bands <200 kDa in SDS-PAGE or Western blots [31,32]. Importantly, the Stab2 cloning report [17] showed no Western or SDS-PAGE data to identify the size or heterogeneity of expressed recombinant protein; thus, HARE was either unnoticed, ignored, or not considered relevant at the time.

The half receptor HARE was confirmed to be relevant and naturally occurring by the demonstration that recombinant human Stab2 expressed in all cell types tested is reproducibly processed to generate twice as much HARE as intact Stab2 [21]. Until the controlled partial cleavage process that creates HARE from Stab2 is inhibited or eliminated in future recombinant cells, the field remains unable to determine and understand any individual binding and endocytic differences or abilities of HARE versus Stab2.

In contrast to endocytosis, phagocytosis mediated by Stab2 is likely not performed effectively by the much smaller HARE, which only has ≤half of the domains (Figure 2) needed for cell engulfment [18,19,20]. A clear functional difference between HARE as a primary SEC endocytic receptor and Stab2 as a primary macrophage phagocytic receptor warrants nomenclature updates [33] that recognize HARE and Stab2 as functionally distinct receptor isoforms.

## 6. All GAG Ligands Bind to HARE, the C-Terminal Stab2 Half-Receptor

### 6.1. Three Other GAG Ligands Were Reported between 1981 and 2003

Figure 3 summarizes the organization of the 8 GAG binding regions, as well as DxS, in the HARE ectodomain. As I noted previously [33], Fraser et al. [34] discovered the first ligand HA in 1981 by its clearance from the blood by rat liver SECs. The HARE binding site for HA was confirmed by Harris and Weigel [26] in 2008 to be the 92 aa Link module (Figure 2 and Figure 3); a HARE variant lacking this region has no HA binding activity. The second GAG ligand was CS-A, reported by two groups in 1986 based on its ability to inhibit HA clearance by the new receptor, [8,35,36]. CDR is the backbone polymer for all the CS variants (Figure 1). Although unmodified CDR is a minor fraction of the CS-GAGs, it was the third GAG ligand reported [8,37]; able to compete up to ~50% for HA binding and endocytosis by HARE. DxS was discovered to be a Stab2/FEEL2 ligand in 2003 by Tamura et al. [38].

DxS is synthesized chemically by sulfation of the neutral branched glucose polysaccharide dextran, which has a linear backbone of α-linked D-glucopyranosyl units. This large mass microbial product is used as a plasma volume expander, a drug administered intravenously. Although dextran is not a mammalian molecule, the finding that highly sulfated DxS is recognized and internalized by HARE/Stab2 is significant, in that it indicates the DxS negative charge distribution patterns mimic those of *bone fide* human ligands. Whether DxS serves as a mimic for some of the known ligands (e.g., HEP, Figure 3) or is a proxy molecule for still unidentified ligands is unknown.

### 6.2. Five Additional GAG Ligands Were Reported in 2008

Hep was identified as a ligand for human HARE, when Harris et al. [39,40] showed that this liver SEC receptor was responsible for its uptake, despite the fact that Hep did not compete for HA uptake by the same receptor. Prior to the molecular cloning in 2002 of HARE by Zhou et al. [41] and Stab2 by Politz et al. [17], the only way that investigators could identify other receptor ligands was if the candidate molecule inhibited the binding and clearance of the first ligand HA. This limitation, of course, meant that a ligand with an independent binding site that did not interfere with HA binding could not be identified.

Thus, earlier studies showing that Hep did not compete for HA binding led to an ultimately incorrect, but understandable, conclusion that Hep was not a ligand. Additionally, prior to the era of molecular biology and for similar reasons, there was little evidence that individual proteins could possess multiple binding activities; as is also true for many enzymes with very specific biochemical activities, binding proteins were generally considered mono-functional (one binding partner).

Amazingly HA, CDR, CS-A and a GAG-mimic (DxS) were the only GAG ligands identified over 26 years (1981–2007) and then, within a year, the number of GAG ligands shown to bind and be cleared by HARE/Stab2 more than doubled, to nine. In 2008 Harris et al. [26] found four other GAGs also bind and are endocytosed by HARE: CS-C, CS-D, CS-E and DS. The initial designation CS-B was later changed to DS, since this GAG contains iduronic rather than glucuronic acid, which is present in all other CDR species (Figure 1). Link-module deletion studies [26] also confirmed that the Link module is responsible for HARE binding to HA, CDR, CS-A, CS-C and CS-D (Figure 3).

### 6.3. Organization of GAG Binding Domains within HARE

HA and the five CDR-GAGs (CDR, CS-A, CS-C, CS-D and CS-E) bind within the relatively small Link module, which is proximal to the membrane domain within the HARE ectodomain (Figure 3, right yellow circle). Inhibition of HA endocytosis by different GAGs in stable cell lines expressing human HARE enabled assignment of their binding domains, and involvement of the Link module [26]. In contrast, we know the Hep binding site is between the Link module and the HARE N-terminus, but the regions within the HARE ectodomain involved in Hep binding (Figure 3, middle yellow circle) are still unidentified. Removal of the Link module does not alter Hep binding or uptake, although the Hep binding region appears to be spatially close to the Link region. CS-E and DS bind within the Hep binding site, partially blocking Hep binding and, to a lesser extent, both also partially inhibit HA binding [26]. A third independent binding site for AcLDL (Figure 3, white circle), presumably on the N-terminal side of the Hep binding site, also binds to both DS and DxS, based on their partial inhibition of both Hep and AcLDL binding. DS effectively blocks Hep binding and DS also partially inhibits both HA and AcLDL binding, apparently spanning the three binding regions (Figure 3, longest gray oval).

### 6.4. HA Binding Is Mediated by a Link Module, also Found in Other Extracellular Proteins

Many biomatrix proteins contain an HA-binding Link module, including aggrecan, brevican, CD44, HARE, link protein, neurocan, Stab-2, TSG-6 (Tumor necrosis factor-Stimulated Gene-6) and versican. However, the presence of a Link module does not mean that protein is able to bind HA. An example is Stab1, in which the Link module does not bind HA [17,30], although the closely related Link module in HARE/Stab2 binds HA with high affinity, a K_d_ = 7 nM [25]. The tertiary structure of the TSG-6 Link module was determined using solution NMR by Kohda et al. [42]. The relatively small HA binding Link modules (<100 aa) contrast greatly with the size of the HA polymers they engage, which range in size from 10 [43] to 50,000 sugars (10 MDa). The human TSG-6 Link module consists of two α-helices and two antiparallel β-sheets arranged around a large hydrophobic core and this structure defines the consensus fold for the Link module superfamily.

In this elegant and important study, Blundell et al. [43] determined the solution structure of the TSG-6 Link module before and after binding to HA, identifying a well-defined open binding groove with bound HA on one face of the Link module. In the absence of HA, however, this groove is closed. Thus, the Link module undergoes a significant conformation change upon groove opening associated with HA binding, substantially altering the arrangement of an external loop within the module. The discovery of this HA-induced conformational change, likely conserved among many Link module superfamily members, is highly significant for understanding how such a structurally simple polysaccharide might activate intracellular signaling.

An HA-dependent conformational change could create a new binding site for an event needed for HARE mediated signaling. Further NMR and calorimetry studies with defined oligosaccharides in this study by the Day group [43] enabled them to estimate the minimum HA length for fully occupying the binding groove, as well as the oligosaccharide polarity in the groove (i.e., the reducing–nonreducing end orientations). Binding constants are essentially identical for HA oligosaccharides with at least 7–10 sugars.

### 6.5. Unlike TSG-6, the HARE Link Module Does Not Enable Simultaneous Binding of HA and Hep

The Hep binding site within HARE is still unidentified and may be situated within the E4, F5 or F6 domains of the protein (Figure 3). Mahoney et al. [44] determined that in TSG-6 the Hep binding site is within a separate binding groove of the Link module, distinct from the HA binding groove. Both HA and Hep can bind to TSG-6 via the Link module but HA and Hep bind within the Link module in separate binding grooves. When Hep binds, there is a conformational change in the protein that prevents HA from binding and likewise, if HA binds then the Hep binding site conformation is changed and inactivated. Thus, HA and Hep cannot bind simultaneously to TSG-6 [44].

In contrast, despite the TSG-6 and HARE Link modules having high sequence identity (48%) and homology (64%), there is no contribution to Hep-binding by the HARE Link module. This is clearly evidenced by the normal Hep binding activity of the HARE(ΔLink) receptor [26], which is as good as the WT receptor. Thus, this different functionality of the HARE versus TSG-6 Link modules, means that both HA and Hep can bind with HARE simultaneously. This dual uptake feature might help optimize the ligand uptake capacity and efficiency of HARE as an SR.

## 7. Both Stab2 and HARE Can Activate Intracellular Signaling

### 7.1. Relatively Few Studies Have Focused on Cell Signaling Mediated by Stab2/HARE

Based on the diverse tissue distribution of HARE/Stab2 found by Zhou et al. [24,28,29] and Falkowski et al. [16] in 2000–2003, I expected that more studies on these SR isoforms would have been reported by mid-2020, over 17 years later. These proteins are highly expressed in the SECs of the liver, spleen, bone marrow and lymph nodes and in specialized structures of the eye, heart, brain, and kidney. Stab2 expression is also found in corneal and lens epithelium, heart valves mesenchymal cells, in the ependymal cells lining brain ventricles and prismatic epithelial cells that cover renal papillae [16].

However, PubMed searches for “Stab2 and signal transduction” (September 2020) identified only 20 publications, since the molecular cloning of Stab2 and HARE in 2002, nine of which (about half) did not directly study Stab2 cellular signaling. For example, Hamoud et al. [45] used a proteomic approach to determine that Stab2 interacts with and activates the G-protein coupled receptor activity of Brain-specific Angiogenesis Inhibitor 3 to stimulate its fusion promoting activity. Fajardo et al. [46] used indirect mouse genetic proteomics to show that calcineurin signaling impairment reduces Stab2 expression.

Similarly, Stab2-Cre mice were used by Leibing et al. [47] to study the effects of knocking out *wnt* signaling by using endothelial subtype-specific Stab2-Cre driver mice to delete the *Wnt* ligand-secretion-mediator gene from liver SECs. These gene manipulations did not alter Stab2 expression. In this, and several similar studies in the above search, the Stab2 gene locus was used to create a tissue cell-specific gene knockout; experiments were not focused on Stab2 function or signaling. Additionally, Koch et al. [48] conditionally deleted bone morphogenetic protein-2 (Bmp2) in mouse liver SECs by also using SEC subtype-specific Stab2-Cre mice. Genetic inactivation of hepatic angiocrine Bmp2 signaling caused a large liver iron overload with elevated serum iron levels and organ iron deposition. Again, this was not a Stab2 signaling study.

Of the 20 Stab2/HARE publications identified above, 11 were signaling or targeted uptake studies or reviews; two are from the Kim group and eight are from the Weigel group. Harris and Cabral [49] also recently reviewed many aspects of cell signaling mediated by Stab2/HARE. Less specific search terms (e.g., Stab2 and signaling) identify about 50% more studies, but these are predominantly ancillary, not involving Stab2-specific signaling.

### 7.2. Stab2 Mediates PS Recognition and Phagocytosis of Apoptotic Cells

Park et al. [50] found that Stab2-mediated phagocytosis of cell corpses requires the adaptor protein GULP as a downstream molecule in the Stab2-mediated signaling pathway, ultimately leading to the release of Transforming Growth Factor-β (TGFβ). The phospho-Tyr (pTyr) binding domain of GULP binds to the NPLY motif in the Stab2/HARE CD. The finding that Stab2 recognizes and senses PS exposed on apoptotic cells as an “eat-me” signal, led Kim et al. [51] to propose that Stab2 binding of PS patches is required for sensing and creating a “fuse-me” signal as the initial indicator to start myoblast fusion. More recently, Penberthy and Ravichandran [52] reviewed how other PS SRs, including Stab2, mediate downstream signaling. Importantly, macrophage phagocytosis of apoptotic cells mediated by Stab2 and other PS SRs elicits systemic anti-inflammatory responses that release cytokines, e.g., TGFβ.

### 7.3. Stab2/HARE Regulates Arterial-Venous Differentiation in Zebrafish

Rost and Sumanas [53] reported that HARE/Stab2 functions in a signal transduction pathway regulating arterial-venous differentiation during zebrafish embryogenesis. Stab2 morpholino-knockdown embryos lacked intersegmental vessels and showed defects in their axial vessel formation. These Stab2 knockdown embryos also showed defects in arterial-venous differentiation and the normal expression of venous markers. Simultaneous knockdown of Stab2 and HA synthase 2, were synergistic, indicating that important HA and Stab2 interactions occur during vasculature formation.

Stab2 morphants also showed reduced phosphorylation of extracellular signal-regulated kinase (ERK) in the arterial progenitors; phospho-ERK1/2 (pERK1/2) is a known transducer of Vascular Endothelial cell Growth Factor signaling, associated with arterial-venous differentiation. Interestingly, this signaling creates a negative feedback loop that represses Stab2 expression. The authors concluded that HARE/Stab2 is part of a novel and important signaling pathway that regulates ERK phosphorylation during the establishment of arterial-venous development.

### 7.4. HARE Expression during Rat Liver Embryogenesis Is Biphasic

**Figure 4:**
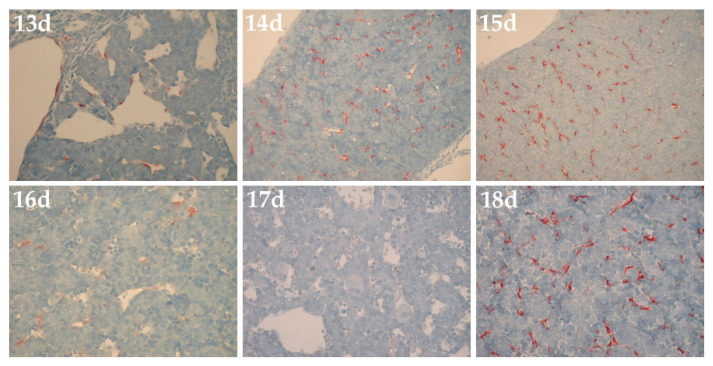
Cycles of HARE expression occur during rat embryonic liver development. HARE/Stab2 protein was detected between day 13 (13d) and day18 (18d) using monoclonal antibodies 30 and 154 raised against purified rat HARE (Magnification 100×). Commercial paraffin tissue blocks (Zyagen) were processed for immunocytochemical staining as described by McGary and co-workers [54,55]. Carl T. McGary, MD PhD is at University of Minnesota Medical School of Medicine-Duluth Campus, Department of Biomedical Sciences, SMED 227, 1035 University Drive, Duluth, MN 55812.

In an unpublished 2001 study to assess HARE expression during fetal development, Dr. Carl McGary (then at the University of Rochester) examined commercial fetal rat liver tissue blocks for immunocytochemical staining with monoclonal antibodies against rat HARE developed in the Weigel group [28,29]. Although these results are not peer-reviewed, I believe it is important and appropriate to share these unpublished data here. My laboratory was closed in 2018 and few investigators are focused on Stab2 and HARE. I hope others may be motivated by these preliminary findings to study HARE/Stab2 expression during embryogenesis (Figure 4) and phosphorylation of the HARE CD (Figure 6), in efforts to refute, confirm or extend these preliminary findings further.

The results showed a complicated pattern of time-dependent change between day-13 and day-18 (Figure 4) with increased expression from day-13 to day-15 but then a rapid decrease of about 90% by day-16 to very sparse staining. No HARE expression was detected on day-17. However, from day-18 until day-21 (birth) even more robust staining was observed in the almost mature sinusoidal LECs. Idiotype matched mouse nonimmune antibodies showed no staining on any day. HARE expression in amniotic membranes was also seen at day-10, but not day-11 or later (not shown). The biphasic HARE expression pattern may be important for several reasons. 

First, it could indicate that extensive, although transient, tissue creation within the embryo is associated with high levels of HA and PG-GAG turnover, with the need for efficient clearance and degradation of the resulting biomolecular trash by an early embryonic scavenger function of newly expressed HARE/Stab2.

Second, a biphasic wave of HARE expression might also indicate a prenatal form of HARE/Stab2, perhaps a splice variant [23], with a novel clearance function (i.e., ligand specificities) not found in the adult proteins. A third scenario could be the occurrence of two separately timed expression patterns for each of two functionally different receptor isoforms (e.g., HARE in one wave and Stab2 in another) or splice variants found in embryos, adults or both. 

Of course, other scenarios might also explain the complex biphasic expression pattern of HARE/Stab2 proteins during mouse embryonic development.

## 8. HARE Mediates Coordinated HA Endocytosis and ERK1/2 Phosphorylation

### 8.1. Free HARE Is in Complexes with Three Different Protein Kinases

The same year that Park et al. [50] reported that a Stab2-mediated signaling pathway is stimulated during phagocytosis of cell corpses, Kyosseva et al. [56] reported that HARE is bound to protein kinases, even in the absence of ligand. Using human Flp-In 293 cells stably expressing either HARE alone or both Stab2 + HARE, inherently generated [21] by proteolysis of full-length Stab2 (Section 5.1), they found that immunoprecipitated HARE was complexed with ERK 1 and ERK 2 (ERK1/2), c-Jun N-terminal protein Kinase (JNK), and p38 protein kinase. It was not determined if one HARE protein is in a complex with multiple signaling kinases or in separate complexes, each with one HARE and an individual signaling kinase.

### 8.2. HARE HA Endocytosis Activates ERK1/2 but Not JNK or p38

Kyosseva et al. [56] also reported that HARE binding and endocytosis of HA initiates an ERK signaling response (i.e., ERK phosphorylation). In contrast to ERK1/2 activation, HA did not induce phosphorylation of JNK or p38 kinases [56]. Perhaps HARE•JNK or HARE•p38 complexes are activated by HARE-mediated endocytosis of other ligands (note that to minimize confusion in describing biomolecular complexes, dash symbols (-) represent covalent bonds and dot symbols (•) represent noncovalent bonds).

They found pERK1/2 increases in dose- and time-dependent manners when HA is added to cells expressing Stab2 + HARE or HARE only; but not in vector-only cells. Maximum pERK1/2 levels occur in 30 min at 10 nM HA, and the response dampens at >40 nM HA. As expected for signaling cascades, the extent of ERK1/2 activation peaked and then decreased after 30 min. HA uptake and ERK1/2 phosphorylation decreased 90% in HARE(ΔLink) cells, confirming that ERK1/2 activation depends on HA binding and endocytosis. HA binding and uptake increased phosphorylation of HARE without increasing the level of preformed HARE•ERK complexes. A relevant result, with respect to the roles of the two receptor isoforms, is that ERK1/2 activation was twice as great in cells expressing HARE compared to cells expressing Stab2 + HARE [56].

### 8.3. HARE•HA Endocytosis Stimulates Tyr Phosphorylation of HARE

**Figure 5:**
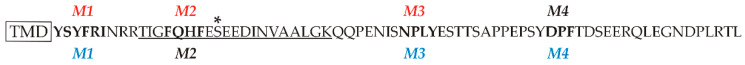
The Stab2/HARE CD has four potential endocytic targeting motifs. The 72 aa CD (Y^2480^–L^2551^) next to the TMD contains four different AP-2/clathrin-mediated targeting motifs (boldface: *M1-M4*) that enable coated pit mediated endocytosis by both receptor isoforms, free or ligand-bound. Motifs in the top row (red) target HARE•HA complexes for endocytosis; *M4* (black) is the only motif that does not mediate HA uptake. Motifs in the bottom row (blue) target HARE•Hep complexes for endocytosis; *M2* (black) is the only motif that does not mediate Hep uptake. A tryptic peptide (underlined) contains phospho-Ser^2497^ (asterisk). Dot symbols (•) denote noncovalent bonding.

Park et al. [50] found in macrophages that the GULP pTyr binding domain binds to the HARE cytoplasmic NPLY motif, indicating that the NPLY domain is phosphorylated and thus a ligand for GULP binding. Consistent with these findings, Kyosseva et al. [56] also reported that HARE endocytosis of HA stimulates phosphorylation of the HARE CD, shown by pTyr-protein immunoprecipitation and detection with anti-HARE Ab and by HARE immunoprecipitation and detection with anti-pTyr Ab. Three of the four Tyr residues in the HARE CD are in endocytic motifs *M1* and *M3* (Figure 5).

Dr. Edward Harris in the Weigel group then performed preliminary studies to determine if purified recombinant human HARE contains phosphorylated regions within the HARE CD. Tryptic digests analyzed using mass spectrometry indicated a potential phosphorylation site within the tryptic CD peptide: TIGFQHFESEEDINVAALGK^2508^ (Figure 5; underlined).

**Figure 6:**
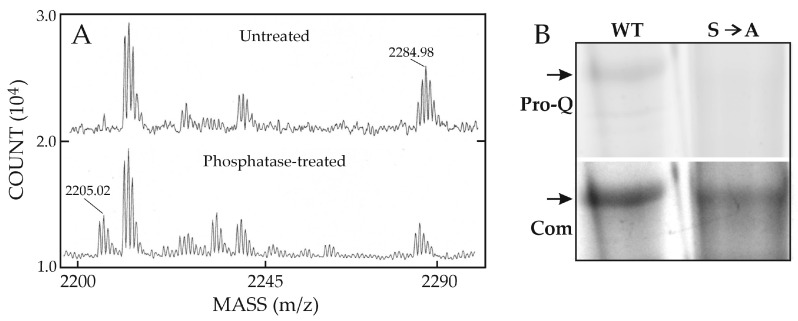
Ser^2497^ is phosphorylated in the HARE CD. (**A**) Immunopurified WT HARE cut from an SDS-PAGE gel was trypsin digested and samples were untreated (top) or treated with alkaline phosphatase (bottom). Mass spectrometry analyses using a Voyager Elite MALDI-TOF instrument in reflector mode revealed signals at *m*/*z* 2284.98 for a phospho-(Thr^2389^-R^2508^) peptide, which was reduced by phosphatase treatment, resulting in strong signals for the predicted free peptide (*m*/*z* 2205.02). (**B**) HARE was immunopurified from stable Flp-in 293 cells expressing WT (left) or S2497A (right) HARE, run on SDS-PAGE, electroblotted, stained with Pro-Q for phosphoryl groups [57,58] and HARE (arrows) was visualized by fluorescence microscopy (top) and then, after washing and staining, with Coomassie blue (bottom).

The expected *m*/*z* signals were found before and after treatment with alkaline phosphatase to remove phosphate (Figure 6). The phospho-peptide was present at the predicted *m*/*z* for one phosphate group; 2285.05 (Figure 6A, top). After alkaline phosphatase treatment, this signal decreased and the free peptide signal (*m*/*z* 2205.08) substantially increased (Figure 6A, bottom). A 79.96 *m*/*z* shift for a phosphoryl group is good evidence this peptide is phosphorylated. 

However, the peptide has two potential phosphorylation sites, at T^2489^ and S^2497^. A mutagenesis study by Harris and Weigel using the sensitive phosphate-specific Pro-Q Diamond^TM^ fluorescent dye [57] showed that Ser^2497^ in full-length WT HARE is phosphorylated in the absence of ligand (Figure 6B, left), whereas a specific-site mutant HARE(S2497A) lacking Ser^2497^ is not phosphorylated (Figure 6B, right). No other CD phosphorylation was evident, indicating that in the absence of ligand, such as HA, Ser^2497^ is the only site phosphorylated in recycling HARE receptors. 

## 9. The Stab2/HARE CD Contains Four Potential Targeting Motifs That Mediate Clathrin-Coated Pit Receptor-Mediated Endocytosis of HA and Hep

### 9.1. HARE Endocytosis of HA Is Mediated by Three of the Four CD Targeting Motifs

Before discussing further aspects of HARE signaling in response to GAG endocytosis, it is important to summarize what has been learned about the four Stab2/HARE CD motifs that could enable targeting of receptors to clathrin-coated pits (Figure 5). To assess the function and relative importance of these four motifs, Pandey et al. [58] performed an extensive mutagenesis study creating multiple cell lines each expressing a HARE deletion mutant with a unique CD lacking one or more of the four motifs: YSYFRI^2485^ (*M1*), FQHF^2495^ (*M2*), NPLY^2519^(*M3*) and DPF^2534^ (*M4*). Not all motifs actively target HARE•HA for endocytosis.

Stably transfected cells expressing HARE(Δ*M1*), HARE(Δ*M2*), or HARE(Δ*M3*), lacking CD motifs 1, 2, or 3, respectively (Figure 5), had decreased HA endocytosis rates (Table 1) of 44–61% compared to WT [58]. In contrast, HARE(Δ*M4*) cells had elevated HA endocytosis rates (119%). Combined deletions of *M1-M2* or *M1-M2-M4* decreased HA endocytosis rates to 61% or 58%, respectively, whereas the HA endocytic rate was decreased to only 7% in mutants lacking all four motifs, *M1–M4*.

HARE(Y2519A) cells expressing a mutated *M3* (NPLY) retained 94% of WT endocytosis, but endocytosis decreased to only 5% of WT when this mutation was combined with the triple motif deletion (Δ*M1M2M4*); this is virtually identical to a mutant lacking all four motifs (Δ*M1M2M3M4*), which was 7% of WT. Tyr in NPLY^2519^ is thus critical for endocytosis mediated via motif *M3*. We concluded that *M3* alone accounts for about 60% of total HA uptake and that *M1 + M2* together account for about 40% of total HA uptake. The highly efficient endocytic route mediated by *M3* absolutely requires the presence of Y^2519^.

An important conclusion is that the maximum rate of HARE endocytosis of HA requires that three of the four endocytic motifs (YSYFRI, FQHF, and NPLY) work together for efficient coated pit targeting and endocytosis of HARE.

### 9.2. Redundant Targeting Motifs Increase Total Ligand Uptake Capacity of HARE by Enabling Parallel Coated Pit Targeting of HARE•Ligand Complexes

Each HARE-specific motif deletant (*M1, M2* and *M3*) showed similar processing and degradation characteristics for the internalized HA, indicating that removing one or several endocytic motifs did not affect ectodomain binding of HA or targeting of internalized HA to lysosomes, mediated by the remaining motifs [58]. We concluded that although NPLY may be the most important motif for HA endocytosis, it functions together with two other endocytic motifs, *M1* and *M2*. Thus, three motif signal sequences, *M1–M3* (YSYFRI, FQHF, and NPLY), provide redundancy and therefore enhanced efficiency and speed for mediating the continuous coated pit targeting and endocytosis of cell surface HARE•HA complexes (dot symbols represent noncovalent bonds).

As a recycling receptor, cell surface HARE is continuously targeted to clathrin-coated pits, subsequently internalized, routed through multiple intracellular endosomal compartments and then returned back to the cell surface [10]. This continuous receptor recycling pathway operates whether HARE is free or bound to a ligand. The presence of three targeting motifs to facilitate HARE•HA endocytosis in the short CD seems highly redundant. This could reflect either the importance of clearing HA effectively or that multiple motifs are used to facilitate the efficient clearance of all the GAG ligands whose turnover is HARE-mediated, or both possibilities could be true.

### 9.3. HARE Endocytosis of Hep Is Mediated Be a Different Subset of 3-Motifs than the 3-Motif Subset Mediating HA Uptake

Pandey et al. [59] assessed the ability to endocytose (Table 1) HA or Hep within a panel of HARE variants with either a single-motif deletion (i.e., three motifs remaining) or containing only a single motif (i.e., three motifs deleted). The *M1, M2*, and *M3* motifs mediate the endocytosis of HA. In contrast, decreased Hep endocytosis was found in single-motif deletion variants lacking *M1, M3*, or *M4*, although the *M3* deletant was inhibited the most; 65% of WT. The remaining functionally redundant motifs did not compensate for the loss of other motifs. Significantly, this group of Hep-competent motifs (*M1, M3, M4*) is a different subset of three motifs than that involved in HA uptake (*M1, M2, M3*), as highlighted in Figure 5.

Surprisingly, a HARE CD variant with only *M3* internalized both HA and Hep, whereas variants with either *M2* or *M4* alone could not endocytose either ligand. Additionally, the internalization of HA and Hep by all HARE CD mutants is dynamin-dependent and inhibited by hyperosmolarity, confirming that the uptake pathway for all the HARE mutants is still via clathrin-mediated endocytosis. These results indicate a complicated relationship among multiple CD motifs that target clathrin-coated pit uptake and a more fundamental and common role for motif *M3*, perhaps related to the phosphorylation of Y^2519^ (Section 11).

## 10. HARE-Mediated Intracellular Signaling Is Stimulated by Endocytosis of only Some GAG Types

Pandey and Weigel [60] assessed signaling using a dual LUC reporter assay [61], in which cells are transfected with two plasmids encoding different LUC genes, one controlled by an NF-κB (Nuclear Factor-kappaB) promoter and the other without a promoter; the ratio of the two LUC signals is then determined in lysates. The system enabled us to determine if HARE-mediated intracellular signaling during the uptake of ligands, including the GAGs, specifically stimulates ERK1/2 phosphorylation that leads to NFκ-B promoter activation and LUC recorder protein synthesis.

### 10.1. Only HA, Hep and DS Uptake Activate HARE-Mediated Signaling

HA, Hep, DS and AcLDL stimulate dose-dependent HARE-mediated NF-κB activation of LUC expression [60]. Half-maximal activation of NF-κB occurrs at low levels of these ligands; 10–25 nM. In contrast, CS-A, CS-C, CS-D, and CS-E do not stimulate NF-κB activation or LUC expression. Confirming that the observed cell signaling response was authentic, we also found that only the four signaling ligands stimulated the degradation of IkB-α, an endogenous NFκ-B inhibitor.

Additionally, testing pairwise combinations of the four signaling ligands showed the activations of NF-κB were additive. Pandey and Weigel [60] also showed that treatment of cells with clathrin siRNA decreased clathrin expression by >50%, which eliminated NF-κB mediated signaling by all four ligands. This finding strongly indicates that HARE GAG complexes that reside on the cell surface are not competent to activate signaling, but that activation of signaling complexes only occurs either (i) during targeting into coated pits, (ii) during endocytosis or (iii) after internalization.

### 10.2. Four Nonsignaling CS Types Block HA Uptake and Signaling

Although the four CS types (CS-A, CS-C, CS-D, CS-E) do not activate signaling [60], they do compete for HA binding to HARE [26]. Thus, these CS competitors of HA binding also effectively block HA-stimulated signaling. Since HA-binding Link modules are found in many PG proteins [43] (Section 6.4), an implication of this latter result is that coordinated turnover of biomatrix HA•PG complexes (with CS chains covalently attached to PG; PG-CS) could have different physiologic outcomes, depending on which of the two GAGs (HA or CS) binds with HARE. An outcome of NF-κB activation, would depend on whether endocytosis of an HA•PG complex occurred via HARE binding to HA, which in turn is bound to the PG protein of the large HA•PG complex. Internalization of HARE•HA•PG-CS complexes formed in this manner could activate signaling. In contrast, the internalization of HARE bound to CS in a HARE•CS-PG•HA complex would not stimulate intracellular signaling. However, based on the HA size-dependent signaling (Section 13) it is likely that many HARE•HA•PG-CS complexes would be too large to enable the Link module and CD changes needed for activation and signaling to occur.

## 11. HARE-Mediated Activation of ERK and NF-κB during HA or Hep Internalization Requires only One of the Four Endocytic Motifs; *M3*

Pandey et al. [62] used NF-κB promoter-driven dual LUC gene assays and stable Flp-In 293 cell lines expressing different HARE CD mutants (e.g., Figure 5 and Table 1) to determine which motifs are needed for Hep-mediated signaling. Single deletions of *M1, M2* or *M4* had no effect on ERK1/2 activation during Hep uptake. In contrast, although Hep uptake still occurs in HARE(Δ*M3*) cells, signaling was eliminated. In cells expressing M3 mutant HARE(Y2519A), ERK1/2 signaling decreased 75%. ERK1/2 signaling was also eliminated in HARE(Y2519A) cells lacking *M1, M2* and *M4* (containing only mutant *M3*). Deletion of *M3* (but not *M1, M2* or *M4*) also inhibited the formation of ERK1/2•HARE•Hep complexes by 67%, in the absence of ligand [62].

NF-κB activation during HARE-mediated uptake of Hep, HA, DS or AcLDL was unaffected in mutants lacking just *M1, M2* or *M4*. However, HARE(Δ*M3*) cells lost HARE-mediated NF-κB activation during uptake of each of these four ligands. NF-κB activation by the four signaling ligands was also abolished in cells expressing HARE(Y2519A) or cells expressing only the site-specific *M3* (NPLY^2519^) mutant; HARE(Δ*M1M2M4*,Y2519A). We concluded that the NPLY^2519^ motif is necessary for both ERK1/2 activation (i.e., pERK1/2 creation) and NF-κB signaling mediated by HARE•Hep endocytosis and that Tyr^2519^ is critical for these functions. Tyr^2519^ is likely important because its phosphorylation is vital in the chain of protein recognition and assembly events during signal activation cascades [50,63].

## 12. HA Activation of HARE Signaling Requires a Link Module N-Glycan

### 12.1. The HARE Link Module N-Glycans Are More Structurally Diverse and Most Are Sialylated, Unlike N-Glycans at Other HARE Glycosylation Sites

HARE contains about 25 kDa of N-glycans, based on the apparent mass losses seen in SDS-PAGE analyses after de-N-glycosylation of purified recombinant rat or human receptor [25,29]. Harris et al. [64], in collaboration with Ann Dell’s group, performed glycoproteomic analyses of purified recombinant human HARE ectodomain and identified a diverse population of branched glycans at 10 of the 17 consensus N-glycosylation sites. The greatest diversity of structures, including the only sialylated glycans, occurred within the HA-binding Link module at Asn^2280^. This site contains 15 bi- or tri-antennate N-glycans, seven of which are sialylated. To determine if these N-glycans are required for HA binding or signaling, we created human Flp-In 293 cell lines expressing membrane-bound or soluble HARE(N2280A) ectodomain variants, unable to N-glycosylate the Link module.

### 12.2. HARE Lacking an N-Glycan in the LINK Module Mediates Normal HA Binding and Endocytosis but Is Unable to Activate NF-κB and LUC Recorder Gene Expression

Membrane-bound HARE lacking Link module N-glycans mediated rapid HA endocytosis but purified HARE (N2280A) ectodomain showed little or no HA binding in ELISA-like HA-HARE pull-down assays or by surface plasmon resonance analysis [64]. The latter analyses detected a very high affinity binding of WT HARE ectodomain to HA (K_d_ = 5.2 nM).

Pandey and Weigel [65] then discovered that stable cell lines expressing HARE(N2280A) bind and endocytose HA, Hep, AcLDL or DS normally. However, no ERK1/2 activation occurred during HA endocytosis by HARE(N2280A) cells, whereas ERK1/2 activation did occur during Hep uptake. Dual-LUC recorder assays showed that increased NFκ-B-mediated gene expression occurred normally in HARE(N2280A) cells endocytosing Hep, AcLDL, or DS but did not occur with HA. In agreement with these data, activation of NFκ-B by endogenous degradation of IκB-α was observed for HARE(N2280A) cells endocytosing Hep, AcLDL, or DS but not HA. We concluded that a Link module complex N-glycan is required specifically for HARE•HA-mediated activation of ERK1/2 and subsequent NFκ-B-mediated gene expression and that this initial activation mechanism is different from and independent of the initial mechanisms for HARE-mediated signaling in response to the endocytosis of Hep, AcLDL, or DS. The Link module N-glycan may thus stabilize interactions that facilitate HA binding to HARE in a manner that activates ERK1/2/ and NF-κB signaling.

## 13. Link Modules in TSG-6, CD44 and LYVE-1 Show Conformational Changes upon Binding HA

Knowing how other Link module proteins function might help to understand how HA binding to the HARE Link module initiates cell signaling. Many structural features are conserved across the Link module superfamily, although important differences occur related to the regulation of HA-binding activity. Unlike the more functionally simple clearance functions mediated by HARE/Stab2, which continuously bind, internalize and destroy ligands, other Link module-containing proteins involved in more complicated biologic functions have mechanisms to turn Link HA-binding activity on or off.

### 13.1. The TSG-6 Link Module

Park et al. [66] reported that when the TSG-6 Link module binds to a short CS oligosaccharide, it induces the dimerization of Link modules. Blundell et al. [67] created and tested molecular models, using NMR spectroscopy to identify the novel features of HA binding to the TSG-6 Link module that confers specificity for HA versus other GAGs. Using sequence alignment among all human Link modules to predict the functionally important HA binding residues, they performed cycles of prediction and NMR testing, and then constructed homology models for how other human Link modules could bind to HA, in silico. These analyses showed that many of the HA TSG-6 binding features are conserved across the Link module superfamily.

These analyses [67], predicted the human HARE/Stab2 Link module would be functionally active, as had been demonstrated earlier with recombinant rat HARE [33,41]; an HA octasaccharide could be modeled bound to the human HARE Link module in the same conformation as found for the TSG- 6 Link module. Importantly, the latter finding means the HARE Link module likely undergoes similar conformational changes as occur when HA binds to TSG-6 [43].

TSG-6, like many other HA binding proteins, contains a consensus Link module in which the fold is comprised of six β-strands and two α-helices held together by two disulfide bonds. CD44 and LYVE-1 consensus Link modules are distinct from the TSG-6 module in that they have extensions composed of four additional β-strands and a third disulfide bridge.

### 13.2. The CD44 Link Module

In collaboration with Tony Day and Martin Noble, David Jackson’s group [68], used X-ray crystallography and NMR to study how changes in CD44 N-glycosylation can switch the receptor “on” or “off” under appropriate conditions. They found that sequences flanking the Link module form a lobular extension to the Link module, creating an enlarged HA binding domain and a novel protein fold. The presence of a glycan at key N-glycosylation sites revealed how specific sugar chains might alter both the affinity and avidity of HA binding to the CD44 Link module, by stabilizing or driving substantial conformational changes in the Link module and flanking regions to activate HA binding activity.

The Day and Jackson collaborating groups also showed [69] that the CD44 Link module binds HA only after appropriate functional activation and that HA binding then induces a conformational transition. Using NMR to assess the structure of the CD44 Link module, they also found it has N- and C-terminal extensions that are not found in the TSG-6 Link module. Takeda et al. [70] reported that HA binding causes β-strands in this lobe to rearrange creating significant conformational changes, including order-to-disorder changes in the adjacent C-terminal end of the module.

### 13.3. The LYVE-1 Link Module

The lymphatic vessel endothelial cell HA receptor-1 (LYVE-1), discovered by the Jackson group [71] is a member of the Link protein superfamily, and is most like the leukocyte HA receptor CD44. Banerji et al. [72] showed that LYVE-1, in common with CD44, contains extended HA-binding regions and a bridging disulfide bond, flanking the N- and C-Link module termini; all these features are critical for HA binding. The soluble monomeric extended CD44 Link module binds HA (K_d_ = 66 µM). Although full-length LYVE-1 ectodomain also binds HA even better (K_d_ = 36 µM), the monomeric extended LYVE-1 Link module itself is inactive, requiring synthetic dimerization for HA binding activity. These results [72] indicate important structural and functional differences between LYVE-1 and CD44, and that adjacent glycans or sequences are needed for high-affinity HA binding by the LYVE-1 Link module.

LYVE-1, like CD44, has a relatively weak unimolecular binding affinity for HA and requires receptor clustering for strong HA binding in lymphatic endothelium. Banerji et al. [73] demonstrated that unlike other HA receptors, LYVE-1 can form homodimers dependent on a specific disulfide bond. In lymphatic endothelium in vitro and in vivo, LYVE-1 homodimers are the predominant species. Dimer formation uses Cys^201^, in each membrane-proximal monomer domain, resulting in HA binding affinity 15-fold greater than the monomer. LYVE-1 mutants unable to dimerize do not bind HA and reduction of the homodimer disulfide bond abolishes HA binding. Small-angle X-ray scattering showed that the disulfide bond forms a hinge that keeps a homodimer in an open scissor-like conformation. The authors proposed that sufficient spatial separation of each Link module within a homodimer allows the same HA chain to bind to both Link modules simultaneously [73].

I note that one dimer binding to two sites on the same HA chain would substantially increase HA binding affinity, but without greater intrinsic affinity needed for either monomer Link site (Section 14.3). Consistent with this interpretation, Banerji et al. found [73] that the higher dimer affinity is largely due to a >60-fold slower off-rate, as opposed to a faster on-rate. This is expected because an on-rate for a sequential 2-step binding process must, a priori, be slower than for a comparable 1-step process (HA and 1 binding partner).

## 14. HA-Binding Protein Interactions with HA: Affinity Versus Avidity

Before discussing the HA size-dependence for intracellular signaling, it is necessary to consider how multivalency affects the nature and strength of binding. The concept and measurement of binding affinity describe two molecules (A and B) coming together to create a transient or long-lasting complex (A•B), depending on the strength of noncovalent interactions between them. In many chemical and biomedical disciplines, an affinity constant (K_d_) for two univalent binding partners forming dimeric complexes at equilibrium is determined and quantified based on the criteria and analyses established by Scatchard [74] in 1949.

### 14.1. There Is No Reason to Believe That the Binding Affinities between HARE and HAs of Various Size Are Much Different than for Other Proteins with a Link Module

Link module binding grooves only have a limited number of possible interactions with the 8–12 sugars involved, regardless of the overall HA length. The K_d_ values for HA 8–12mer oligosaccharides that completely occupy a Link module binding grove are in the µM range [36,43] which is weak, only lasting a few mins before dissociation. In contrast, K_d_ values for larger HA binding to Link-module proteins decrease in direct proportion to HA length (mass). For example, our HARE/Stab2 HA binding studies utilizing ≤100 kDa HA preparations found much higher affinity interactions, with nM K_d_ values [25,37,64]. Laurent et al. [36] examined SEC binding (via HARE/Stab2) to HA, ranging in size from 4 to ~32,000 sugars. They reported similar µM K_d_ values for HA 8–12mers as found by Blundell [43]. However, binding to a much longer 6.4 × 10^6^ kDa HA [36] was about a million-times stronger with a K_d_ value of 9 pM.

### 14.2. Apparent High Affinities Are Due to Great Avidity Effects for a Link Module-Protein Interacting with One Long HA Chain

The avidity concept originated in immunology to indicate and explain very strong binding interactions between antibodies and their target antigens, particularly if either is multivalent or abundant on cell surfaces. Avidity describes the total binding strength of a multi-component complex, including all the noncovalent interactions between A and B sites. It includes the binding affinity of a single complex, the valency of each partner, A and B, and the overall number of A•B interactions comprising the complexes of all binding partners.

Avidity effects become extremely complex and strong for HA interactions with cell surface receptors, especially if the receptor forms multivalent clusters. Such avidity effects have been well studied for CD44 by several groups, most notably the David Jackson and Tony Day groups [68,69,75,76,77,78]. HA is likely the most polyvalent extracellular mammalian molecule with the most specific binding partners, and it is ubiquitous throughout tissue biomatrices. If one HA binding site is 12 sugars (~2.4 kDa), then each MDa of HA (~5000 sugars) has ~415 nonoverlapping binding sites. With overlap, keeping a disaccharide register, this increases 6-fold to 2490 binding sites/MDa.

### 14.3. A Major HA Avidity Effect Is Due to Ultra-High HA Binding Site Concentrations Enabling Rapid Recapture of the Same Dissociated HA Chain by a Link-Module Protein

Small shorter HA has an extended, rod-like secondary structure, whereas larger HA in solution is not rod-like. Larger HA is a random coil with a well-defined size, which for 1.2 MDa HA chains is >200 nm, 0.2 µm [79]. To put this size in perspective, it is larger than most viruses, similar in size to the smallest bacteria or a layer of endoplasmic reticulum, but it is not visible by light microscopy (i.e., it is shorter than visible light wavelengths). Although an average HA size (diameter) can be measured, HA is nonetheless dynamic with regular movements of chain helical segments (e.g., bending, straightening) within the same molecule. Over time, the average hydrodynamic size and shape of 1.2 MDa HA is a 0.2 µm diameter particle.

The volume of a 0.00002 cm (0.2 µm) diameter HA molecule is (4/3)(π)(1 × 10^−5^)^3^ cm^3^ (mL) which is 4.19 × 10^−15^ cm^3^ or 4.19 × 10^−18^ L. The 2490 potential Link binding sites within one 1.2 MDa HA molecule represent 2490 ÷ (6.023 × 10^23^) = 4.13 × 10^−21^ mole of binding sites. These mole and volume values yield a Link module binding site concentration of (4.13 × 10^−21^ mol) ÷ (4.19 × 10^−18^ L) = 1 mM! This is a high concentration of Link HA-binding sites relative to concentrations of most other biomolecules in the local environment. In fact, MDa HA cannot be dissolved to make a 1 mM solution in the lab. It is too viscous and gel-like to stir and virtually impossible to avoid shearing into smaller HA fragments that are more readily dissolved [7,79,80].

The avidity effect of HA binding to a Link-module protein can be understood based on the very high probability that a dissociated HA chain is quickly recaptured by the same membrane-bound Link module protein before the volume-elements of the two partners disentangle and fully separate. Although an HA chain might initially be captured by a Link module binding to a peripheral site on a spheroid-like HA particle, random movement of the bound HA (with more degrees of freedom than the membrane-bound protein) could rapidly surround the protein. Once the protein is within the HA chain volume-space, then the high concentration of surrounding Link binding sites essentially ensures that dissociated HA rebinds to the protein before the HA chain “lifts” away from the protein. A cartoon version might show a person standing on a moving train car with a package in one hand and the other hand holding a strap hanging from the ceiling. If they let go when the train lurches and reach up to grab another, they will be more successful if multiple straps are hanging in the same area.

## 15. HARE-Mediated Signaling in Response to HA Endocytosis Is Highly Dependent on HA Size

HARE binds and clears 14 different soluble ligands [33], of which 8 are GAGs (Figure 3), via clathrin-mediated endocytosis, and HA accounts for a major portion of the biomass turned over by this systemic pathway. HA is also the most polydisperse biomolecule in mammals [12,13], found in vivo ranging in size from short (<2 kD) oligosaccharides [81] to very long polymers (≥10 MDa).

### 15.1. Only Uptake of HA in the 40–400 kDa Mass Range Activates HARE Signaling.

**Figure 7:**
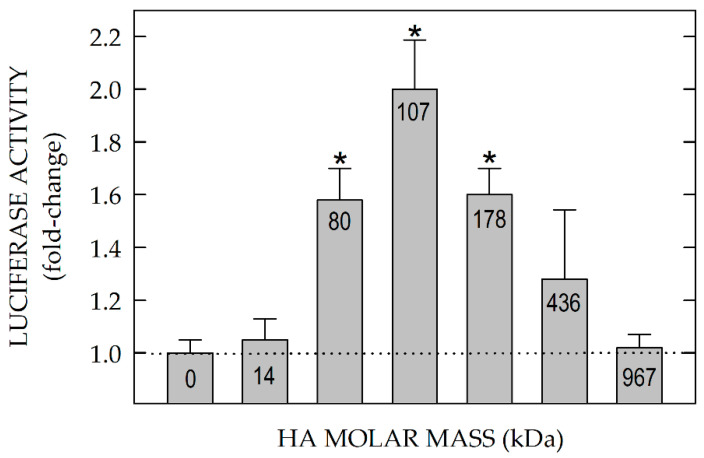
Cell signaling in response to HARE-mediated HA endocytosis is highly dependent on HA size. Specially prepared narrow-size range HAs of different mass (14–967 kDa; 0 is no HA), as indicated in each bar, were tested for the ability to activate signaling in cells expressing HARE, using a dual LUC reporter gene assay [81]. Identically treated empty-vector cells were at a ratio ≤1.0 (dotted line), indicating no NF-κB promoter-specific signaling (*, *p* < 0.05).

Pandey et al. [80] assessed the HA size-dependence for HARE-mediated signaling (Figure 7) by testing purified HA fractions, each with narrow size distribution and different weight-average molar mass, for their ability to stimulate human HARE-mediated gene expression, using an NF-κB promoter-driven dual LUC reporter system. ERK1/2 activation was very HA size-dependent. In contrast to the stimulatory behavior of 40–400 kDa HA, signaling and gene activation were not detected with oligomeric or small HA, <40 kDa. 

More surprisingly perhaps, no signaling occurred with HA >0.4 MDa. Endogenous NF-κB activation also occurs in the absence of transfection with LUC plasmids, as assessed by the expected degradation of IκB-α. HARE•HA mediated activation of signaling during endocytosis was eliminated by deletion of the HA-binding Link module and by a specific HA-blocking anti-HARE monoclonal antibody [29]. This antibody is effective in several types of in vitro assays and shows highly specific inhibition of HA uptake by liver SECs in situ, in perfused rat liver [82].

The ability of multiple Link module proteins to bind to the same HA molecule and also to interact stably with each other is very dependent on HA mass (size), since HA is multivalent with respect to the small number of sugar residues required for binding (Section 14). Blundell et al. [43] found that an 8-mer was an optimal size HA fragment able to occupy the TSG-6 HA Link module binding groove fully and a 12-mer fragment appeared even better in terms of apparent affinity. For comparison, a 10 kDa HA chain has about 50 sugars, so this length chain contains 14 overlapping Link module binding sites with identical reducing end polarity and disaccharide register.

### 15.2. Larger or Smaller HA or Excess Signaling HA Block the Ability of 40–400 kDa HA to Activate HARE Signal Transduction

The HA size-dependent signaling responses occurred similarly for cells expressing either human or rat HARE [80]. Importantly, since HA of any size can bind and block any other size HA internalized by HARE (with varying efficiency depending on the relative sizes of competing species), LUC gene activation by 40–400 kDa HA is inhibited by both excess smaller or larger HA that is nonsignaling [80]. The typical broad size distribution of HA found normally during homeostasis [12,13] or purified from animal sources does not activate HARE-mediated signaling [80]. 

Importantly, the ability of 40–400 kDa HA to activate signaling is also similarly lost as HA concentration increases. The results, particularly the latter biphasic stimulation of HA, support an activation model in which one HA of appropriate size binds two (or more) HARE Link modules enabling their interaction. Signal competence is lost as the HA concentration increases because the probability of two HAREs binding to the same HA chain decreases.

## 16. A Model for HA-Size Regulation of HARE-Mediated Signaling during Endocytosis

This section necessarily has more conjecture and speculation in considering possible mechanisms that might enable a molecular basis for the very specific activation of signaling by only a narrow range of HA sizes. Although hypothetical, it is a sincere attempt to envision mechanisms that might achieve such a molecularly “elegant” response.

### 16.1. The Central Premise

In unpublished studies, we were unable to find evidence that HARE forms dimers or higher oligomers in live rat LECs or stable human Flp-in cell lines, and to our knowledge, no other group has reported differently. Importantly, therefore, HARE/Stab2 are likely present in vivo normally as a monomer (in the absence of ligand). A core concept of any signaling model is that HA binding to Link modules causes significant conformation change in the HARE CD, which triggers the activation of intracellular signal transduction. Changes that expose or create new binding sites and interactions leading to CD activation might occur actively or passively.

1. The same HA chain could bind to two HARE ectodomains, inducing conformational changes in both that enable stable dimer formation, if the HA chain length is a suitable length to bring the two CDs together, with or without additional TMD or CD conformational changes.
2. Two HARE ectodomains, and thus their CDs, come together upon binding the same appropriate-size HA chain to create a stable HARE dimer, with no conformational changes required. An appropriate connecting length HA might stabilize weak TMD•TMD or intracellular CD•CD interactions that would not otherwise be strong enough to keep the dimer together.

**Figure 8:**
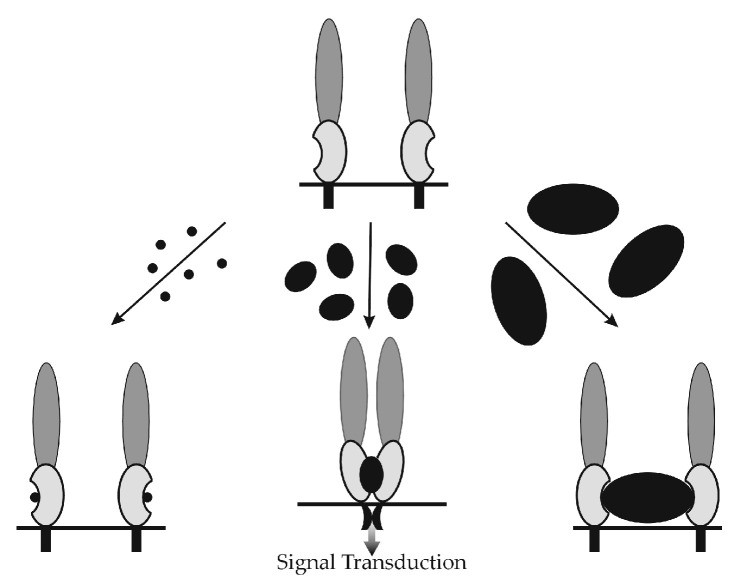
A model for HA-size dependent activation of HARE and ERK signaling. The scheme [80] leads to HARE dimer formation and activation of signal transduction (bottom middle). Symbols denote; membrane (horizontal black lines), CD (black rectangles), HA binding Link module (light gray indented ovals), ectodomain (N-terminal to Link module; long dark gray ovals) and HA size ranges of <40 kDa (small black dots), 40–400 kDa (small black ovals) and >400 kDa (large black ovals).

Either above active (1) or passive (2) scenario might initiate CD activation in response to HA binding to the HARE ectodomain (Figure 8), although Link module conformational changes [43,73] are likely to be involved. Based on the biphasic HA size dependence [80] for signaling (Figure 7), it is very unlikely that HA binding to the ectodomain creates a new binding site within a monomeric HARE CD via a TMD-induced conformational change of the intracellular CD.

In contrast, a process that brings two HARE CDs together to form a dimer would necessarily create new interfaces (potential binding sites) with which a specific signal activation protein (e.g., kinase) could then interact in a manner not possible with a monomeric protein and CD. For multiple reasons noted below, scenario (1) is the more likely mechanism.

### 16.2. Why Is HARE Binding to Small HA Unable to Create Signaling-Competent HARE•HA Complexes?

In the size range of small HA, <40 kDa (small black dots, Figure 8, left arrow), an HA chain could be long enough to bind one HARE but too small, at some lengths, to enable binding of two HARE Link module binding sites to the same small HA chain (Figure 8, bottom left). In order to bring two HARE•HA complexes together in close enough proximity to form a stable dimeric complex, an HA chain needs to be long enough that each Link module fully engages 12–14 sugars with a modest binding affinity [43]. The chain must also contain an optimum range of sugar residues between and bridging these two bound HA sequences to allow correct spatial orientation (e.g., polarity) and distance between monomers so that stable HARE•HARE dimer formation occurs.

Optimal bridging lengths of connecting HA are expected to be within a relatively narrow size range. A connecting HA segment bound to Link modules at each end also likely has adjoining or dangling portions of the same HA chain on the other side of both modules (as for a barbell passed through the center of two weights to create one middle and two extended sections of the barbell).

An under-appreciated aspect of HA structure is that it has, just as for proteins, secondary and tertiary structure [83]. Like proteins, HA polymers form H-bonds between adjacent sugars within the chain. HA forms very stable helices with multiple H-bonds between disaccharide units that create a rod-like secondary structure. Therefore, shorter HA does not readily flex and bend, which can explain why a lower mass HA is unlikely to enable two HAREs bound to the same chain to move in space with the HA curvature needed to bring the proteins close enough to form a dimer.

### 16.3. Why Is HARE Binding to Large HA Unable to Create Signaling-Competent HARE•HA Complexes?

Large HA (>400 kDa; Figure 8, right arrow) is readily able to bind to two or more HARE proteins, although with lower probability as HA chain length increases that they will be proximal (as they move within the radius of the complex). HARE binding to an HA polysaccharide that is 32,000 sugars long (6.4 × 10^6^ Da) has an apparent K_d_ = 9 × 10^−12^ M, which is exceedingly stable with minimal dissociation over days [36]. One such HA chain contains ~1.6 × 10^4^ octasaccharide sequences, in the correct disaccharide register. This high HA multivalency creates great avidity and large cumulative binding energies (Section 14).

With large HA very few, if any, HARE•HA complexes within the same large HA chain would be in close enough proximity to enable their CDs to interact stably (Figure 8, bottom right). Even if two HARE•HA complexes bound to the same long HA chain come together, the binding energy of dimer formation might be offset by the entropy loss of restricting a long section of connecting HA, destabilizing these HARE-HARE interactions. Thus, there would be optimal HA connecting lengths between monomers that enable stable HARE dimer formation.

Dissociation of this bound HARE from MDa HA will be observed experimentally only rarely before the protein rebinds to another site in the same HA chain (Section 14.3). True dissociation of HARE only occurs if the HA chain volume-element diffuses away from the membrane-associated Link module. HARE recapture of the same HA chain becomes more and more likely as HA length, and thus the number of available HARE binding sites, increases. Escape of MDa HA by complete dissociation would occur very infrequently after the initial HARE binding event.

### 16.4. How Could Intermediate Size HA Control HARE•HA Complex Signaling Potential?

In contrast to smaller or larger HA, within the intermediate size range of 40–400 kDa HA chains are long enough to facilitate both high-affinity HA binding to HARE and high-efficiency HARE•HARE binding to occur after two HARE proteins bind to the same HA chain of optimal length. Individual HARE•HA_12_ complexes are then essentially caught (trapped) and the two complexes bound to the same HA chain can only diffuse within the smaller volume, defined by the trimeric HA•(HARE)_2_ complexes. In this confined space, two HARE•HA complexes are more likely and able to interact with each other to create a new dimeric receptor complex. Thus, the formation of a --HA_12_•HARE•[HA]•HARE•HA_12_-- trimeric super-complex should become likely for an optimal HA polymer length (Figure 8, mid-bottom). In the --_12_HA•HARE•[HA]•HARE•HA_12_-- complex, the HA in brackets represents the portion of the bound HA fragment between the two flanking HA 12-mer regions that are bound to HARE Link modules. The [HA] is the intervening and connecting saccharide length between the two HA 12-mer sequences in the same HA chain that connects Link modules bound at random locations along the HA chain. The two free dangling HA ends of the bound chain are denoted by the double dashes (--).

### 16.5. New HARE•HA Dimer Complexes Could Spontaneously Rearrange to Become More Stable

A less optimally stable initial --HA_12_•HARE•[HA]•HARE•HA_12_-- complex is likely to become more stable for two reasons.

First: any of the three possible random single dissociation events (out of two HARE•HA_12_-- and one HARE•HARE) within a 3-partner complex does not enable the “freed” partners to diffuse away, since each is still bound to at least one partner. This essentially ensures that two briefly separated partners can rebind. In the 16.3 example, a µM K_d_ for each HARE•HA_12_ unit becomes a hyper-stable --HA_12_•HARE•[HA]•HARE•HA_12_-- complex with a pM binding K_d_; an extremely high affinity. However, each single µM K_d_ HARE HA_12_-- complex interaction can still dissociate in a few min.

Second: after HARE•HA_12_-- dissociation occurs to give free HARE + HA, a subsequent rebinding event that again forms the full complex can be of greater or lesser binding energy. This situation is self-selecting for creating stronger binding, because when such very stable complexes form randomly by rebinding of a partial HARE•[HA]•HARE•HA_12_-- complex to another HA-12mer (to recreate --HA_12_•HARE•[HA]•HARE•HA_12_--) with an optimal [HA] connecting length, then the new signaling complex may not dissociate again within its functional lifetime.

## 17. The Size-Dependence of HA Signal Activation Is Related to HA Link-Module Orientation in a Dimer Complex

### 17.1. Link Modules Could Form --HA_12_•HARE•[HA]•HARE•HA_12_-- Dimers in Two Orientations

There are multiple ways, with respect to their HA•Link module orientations, for how two HARE•HA_12_ complexes bound to one HA can come together to form a membrane-bound HARE dimer. For example, the bound HA reducing ends could be parallel or anti-parallel (HARE•HARE or HARE•ERAH, respectively), reflecting differences in the degree of each Link module’s rotation about an axis perpendicular to the membrane.

Since HA is bound to a Link module with a fixed polarity [43,69,70], the reducing end can only fit into the binding groove in one of the two possible directions and two possible sugar registers. A short 5–8 sugar HA segment is bound by the Link module if the HA has both a correct sugar (of the two) and end orientation (reducing or nonreducing) to occupy the first sugar-binding site in the HA binding groove [43]. Further complexity in dimer formation and organization is that each group of potential dimer partners (anti-parallel or parallel) can be bound together at many possible regions of their ectodomain circumferences. The Link module orientations, relative to each other, will depend on where the two proteins come together, relative to their Link modules. In a parallel organization with respect to bound HA polarity, the reducing end of HA chain segments bound to both Link modules “point” in the same direction, and the modules can be side by side. In this parallel orientation, the connecting HA chain could be shorter.

However, if the two Link modules face away from each other on opposite sides of the dimer, then these anti-parallel bound HA segments are further away and a longer connecting HA loop is needed depending on the rotation angle between the two Link modules; where around the surface circumference of each protein the HARE•HARE binding occurs.

### 17.2. An HA Rod-Coil Equilibrium Occurs in the 100–250 kDa Size Range

Weigel and Baggenstoss [79] used size exclusion chromatography coupled with multi-angle laser light scattering to determine the weight-average molar mass and diameter of ~60 very narrow size HA preparations ranging in size from 29 to 1650 kDa. We found that the ratios of HA mass:diameter show a clear transition, a break, at a size range of 150–250 kDa (~65–75 nm); with an inflection point at ~175 kDa.

This HA rod-to-coil transition occurs in the optimal HA size range [80] that activates receptor signaling (Figure 7); the mid-point of which is ~50–250 kDa (Figure 7). Since the rod and coil conformational states are both present in the 100–250 kDa size range, they are in equilibrium and at essentially equal concentrations. In contrast, as chains become smaller than ~50 kDa, then the equilibrium favors HA chains being more rod-like and as chains become larger than ~300 kDa, the equilibrium favors more HA chains being increasingly coil-like and globular (e.g., ovoid, elliptical, spherical). Since both conformers are at equilibrium, a 100–250 kD HA chain can be rod-like ~50% of the time and globular ~50% of the time.

### 17.3. The Rod-Coil Equilibrium Might Facilitate Formation of HARE•HA Signaling Complexes

It is likely not a coincidence that the optimal HA length for signal activation is in the size range that allows both secondary structural features to exist simultaneously in different regions of the HA polymer and yet also short enough to ensure the connecting length between bound 12mer segments does not decrease the probability of forming or stabilizing dimeric complexes. Thus, size-specific signaling could be due to unique favorable energetics of some HA regions being able to bind to Link modules in extended rod-like conformations, while connecting regions of the HA chain might simultaneously adopt a more flexible conformation allowing curvature of the connecting HA.

Optimal-size HA for this rod↔coil transition could enable two receptors to bind the same HA, creating a new conformationally altered HARE•HARE dimer that in turn drives the formation of new internal CD•CD dimers to become signal-competent [79]. Creation of a productive three partner complex (2 Link modules and 1 HA) that brings the 2 Link modules close enough in space to form a complex might require that the connecting HA chain be able to bend and loop back on itself; to make a 180-degree turn. This molecular twisting ability would be facilitated at the rod–coil transition sizes, since some HA size ranges will be large enough to have portions of the chain be in either state (e.g., flexible, but like a stiff rope that bends only by having a large bend curvature; not a tight fold).

The two Link-bound HA segments would likely be rod-like, whereas a portion of the connecting HA length must be coil-like in order for the chain to loop around to an anti-parallel or parallel orientation that enables the Link modules to come together. Shorter HA that is almost exclusively rod-like cannot do this. Longer HA can do it over a much larger range of sizes, but most of these will be too long so that even if two bound Link modules form a complex, it will be unstable and only transient.

Our results [80] show (Figure 7) that HARE-mediated signaling only occurs with HA polymer lengths between about 225 (45 kDa) and 2000 (400 kDa) sugars. The most efficient HA lengths for ≥ half-maximal signal activation are ~250 (50 kDa) to 1250 (250 kDa) sugars long.

### 17.4. An Antiparallel, Rather than Parallel, HA Orientation for Dimeric HARE Link-Modules Likely Enables Size-Dependent Signal Activation

The optimal HA length for HARE signal activation by HA is ~140 kDa [80] or ~700 sugars, which is 29-times greater than the 24 sugars engaged by two Link modules in a dimer [43]. I anticipate that the HA_12_ segments bound to the two Link modules in a HARE dimer must be in an antiparallel orientation. The connecting HA lengths between the two modules would be a large fraction of the remaining 676 sugars, and in a relatively broad range of sizes, with varying dangling HA lengths beyond the complex. Many unique active (HA•HARE)_2_ complexes would be created, each with a different HA connecting length (in disaccharide register).

An important point is that different HA connecting lengths would be needed for dimer formation depending on the HA orientation. As noted in 17.1, if the two HA 12mers bound to the Link modules are in a parallel orientation, then they can be side-by-side facing the same direction and the connecting HA length can be relatively short. Even if a longer connecting HA is required it would likely be optimal at a shorter (<700) rod–coil transition size range (e.g., 50–100 sugars) that could facilitate the creation of a smaller bend or loop between two rod-like 12mer segments bound to the dimer Link modules. In this case, an optimal HA length of 700 sugars is inconsistent with two Link modules forming a dimer with a parallel HA chain orientation.

In contrast, a longer connecting HA length is needed for an antiparallel HA orientation for a dimer in which the Link domains face away from each other on opposite sides of the dimer. Perhaps future modeling studies, though theoretical, could corroborate this idea as a possible mechanism for HA size-dependent dimer formation by Link module containing proteins, such as HARE.

## 18. Link Module Conformational Changes upon Binding HA May Be Enhanced by a Link Module N-Glycan That Initiates a Mechanism for HA-Size Dependent Signaling

Signaling during HARE•HA uptake requires N-glycan-dependent conformational changes. The loss of signaling during HA endocytosis in HARE mutants lacking a Link module N-glycan is a surprising finding (Section 12.2). Loss of the glycan at N^2280^ does not alter HARE mediated HA clearance, but signal transduction is eliminated [65]. This loss is very specific for HA-mediated signaling, since HARE(N2280A) cells still retain normal ERK1/2 and NF-κB activation in response to the uptake of Hep, DS and AcLDL. The N-glycan effect on signaling is clearly important to understanding how HARE activation of ERK1/2 is coupled to HA binding and endocytosis. It should be noted that these signaling results were obtained for HARE, and a similar N-glycan-dependent HA signaling for full-length Stab2 has not yet been studied.

### 18.1. A Subset of Proteins with Link Modules Have an N-Glycosylation Site Near Their C-Terminal End

The HARE Link module N-glycosylation site N^2280^KS is 11 aa from the C-terminus of the module, and consensus N-glycan sites are also near this position in the Link modules of TSG-6, CD44 and aggrecan-1 [43]. For example, in the TSG-6 N-glycan site N^118^KS, a glycan would be 13 aa from the module C-terminus. However, the structural studies were with an unglycosylated Link module, expressed in *E. coli*. Unfortunately, it is generally necessary to omit N- or O-glycans for protein structure determinations, due to the great heterogeneity of native glycans at almost every glycosylation site. For example, human HARE ectodomain has 10 N-glycan sites with 4–15 different structures at each site; a total of 72 different bi, tri and tetra-antennae species [64], resulting in a huge number of different combinations and individual glycoprotein structures (not including O-glycans). At least 15 different N-glycans were identified at the HARE Link module N^2280^ site. Although we know an N-glycan is required for signaling, this does not mean that all of the identified glycans may be suitable to ensure this functionality. Each has unique structural features and only a subset of glycans might enable signaling competence. 

Curiously, the aa sequence DAY occurs in Link modules between the N-glycan site and C-terminus in TSG-6, versican-2 and neurocan-2, but not in HARE. In an ironic, yet fitting coincidence, the Tony Day group has extensively studied the structure, function and biology of TSG-6, with particular focus on the Link module, in which the aa sequence DAY is near its C-terminus.

### 18.2. Signal Activation Is Consistent with Conformational Changes upon HA Binding That Induce Intracellular CD-CD Conformation Changes (15.1)

Human Link modules are found in CD44, Lyve-1, Stab1, Stab2/HAARE, KIAA0527, HAPLN1, HAPLN2, HAPLN3, HAPLN4, aggrecan, brevican, neurocan, and versican. Most of our understanding of Link structure and HA binding has been contributed by the Day and Jackson groups [42,43,68,69]. HA binding to the TSG-6 Link module creates a conformational change [43]. Link module structures of CD44 [68,69] and LYVE-1 [71,72,73] have also been determined, and it is likely that HA binding also induces similar conformational changes in those with an N-glycosylation sequon (NXS/T), based on their high level of sequence similarity and identity [43,44]. Such a change might also occur in Link modules containing an N-glycan near the C-terminus, as noted above, but this has not yet been assessed. It is clear, however, that the HARE Link module N^2280^-glycan is required for signal activation, independent of HA endocytosis [65].

### 18.3. A Plausible Scenario

A Link glycan may be needed for conformational changes significant enough, upon HA binding, to alter the HARE dimer TMDs and CDs leading to activation and stimulation of ERK1/2 phosphorylation (Figure 8). The demonstrated Link module conformational change [43,44] could be enhanced or augmented by further conformational changes stabilized by the N-glycan; these coordinated Link module conformational changes could in turn drive a transmembrane CD conformational change to activate signaling. 

Whatever the driving forces, the altered HARE CD regions would then be recognized by a kinase that phosphorylates Tyr^2519^ in *M3* (Figure 5) and that phosphorylates and activates ERK1/2, already bound to the CD and primed for activation (Figure 8, bottom middle; signal transduction arrow).

## 19. Two Stab2/HARE-Expressing Cell Types May Constitute a Systemic Mechanism for Monitoring Biomatrix Turnover and Health

### 19.1. A Tissue-Stress Sensor System

**Figure 9:**
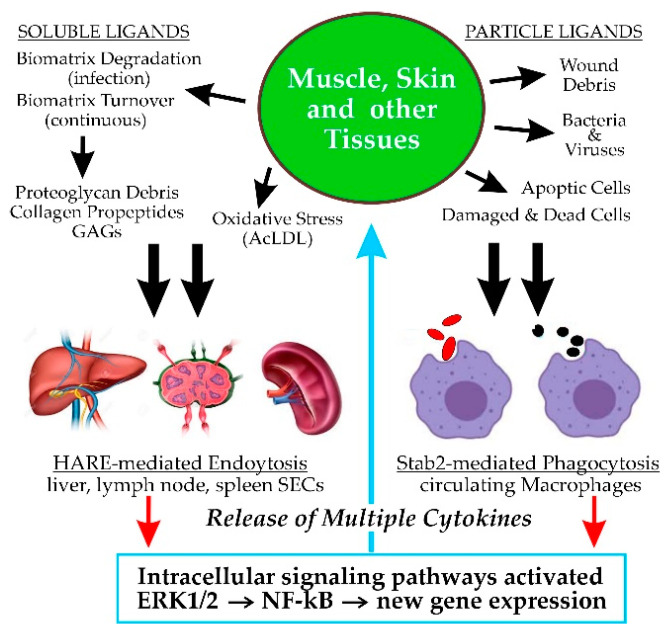
A HARE/Stab2 Tissue-Stress Sensor System. The scheme [84] shows parallel systemic loops in which intracellular signaling is stimulated by HARE-mediated endocytosis (left) of four key soluble ligands (HA, Hep, DS and AcLDL) or by Stab2-mediated phagocytosis of particulate debris or cells (right). These signaling cascades activate genes regulated by NF-κB promoters (red arrows) stimulating synthesis and secretion of pro-inflammatory cytokines such as Transforming Growth Factor-β (TGFβ) [18,50] that can direct new tissue responses (blue arrows) to the physiologic or homeostatic threats [85,86,87]. Soluble ligands shed from body tissues during infection, necrosis or biomatrix turnover are endocytosed by fixed SECs in the liver, lymph nodes and spleen (left). Particle phagocytosis by circulating macrophages (right) removes and destroys apoptotic red or white blood cells (red ovals), wound debris and bacterial cells (black ovals).

Weigel et al. [84] suggested that Stab2 and HARE are part of a systemic feedback system that monitors tissue-stress (Figure 9) and responds to abnormal tissue biomatrix turnover or damage as a danger signal. A central idea is that a subset of ligands serves as indicator signaling ligands that reflect the homeostatsis, whether normal or pathological, of tissue cells and biomatrix components.

The intracellular NFκ-B activation and consequent NF-κB promotor-driven gene expression in response to HARE-mediated endocytosis of HA, Hep or DS support a model in which these responses are part of a bio-monitoring system evolved to obtain an integrated assessment of tissue biomatrix integrity, and overall health. We proposed [84] that the signaling subset of ligands are reporter or indicator ligands that operate in a feedback loop (Figure 9) to monitor and direct responses to a change in the health or distress of systemic tissues and organs. In particular, the key SEC and macrophage responses activate NF-κB promotor-driven gene expression leading to secretion of TGFβ, and likely other proinflammatory cytokines, that would direct more immune cells to sites of tissue damage or stress [88,89,90].

In this Stress Sensor System, full-length Stab2 enables circulating macrophages to bind PS and phagocytose and destroy dead or dying red and white blood cells and debris, as well as Gram-negative and Gram-positive bacteria. HARE, predominantly expressed in fixed SECs of liver, spleen, lymph nodes and perhaps bone marrow clears and destroys soluble molecular components of biomatrices released into the lymphatic and vascular circulations, during either normal homeostatic turnover or abnormal stresses such as wounds and infections.

### 19.2. TGFβ Is Released during Stab2-Mediated Phagocytosis

Macrophage ingestion of apoptotic cells inhibits the production of proinflammatory cytokines through autocrine/paracrine mechanisms involving TGFβ [52,85]. Consistent with this, Huynh et al. [86] showed that the PS-dependent phagocytosis of apoptotic cells stimulates the production of anti-inflammatory cytokines, including TGFβ. Park et al. [18] confirmed that TGFβ is produced and secreted by human monocyte-derived macrophages during Stab2 engagement and phagocytosis of aged red blood cells or PS-containing liposomes. Macrophages exposed to an anti-Stab2 monoclonal antibody were also stimulated to synthesize and secrete TGFβ. Similar increased TGFβ production results were also obtained for multiple assays using stably transfected L-cell expressing Stab-2 [18].

### 19.3. Indicator Ligands

Only a subset of ligands activates intracellular signaling and gene expression. As of 2020, 23 different HARE/Stab2 ligands had been identified [33] that can be classified into three groups: (i) four cell types, (ii) seven modified or cleaved proteins and (iii) 12 anionic polymers, including eight GAGs and four synthetic nucleic acids or polysaccharides (two each). A common characteristic of all ligands is that they are anionic, many with a very high density negative charge and all with spatially distinct negative charge patterns.

An unexpected outcome of the Pandey and Weigel study [60] was that only three of the 8 GAG ligands examined activate signaling (HA, Hep and DS), whereas four do not (CS-A, CS-C, CS-D and CS-E). We also found that HARE-mediated endocytosis of modified LDL (AcLDL) also activates signaling. Many questions still remain, including what downstream gene products are synthesized in response to NF-κB activation.

### 19.4. Why Might DS and Hep also Signal in a Tissue-Stress Sensor System?

HA as a member of this group makes sense in terms of its almost ubiquitous distribution, high levels and very large size. If a driving force in the evolution of a biomatrix monitoring system was the ability to provide an assessment of overall biomatrix integrity in almost all types of body tissues, then it makes sense that several ligands might be potential markers for monitoring the breakdown of diverse types of biomatrices. Hep is made in the liver, lung, other tissues and by circulating basophils and mast cells. Hep is made as a PG (Figure 1) with Hep chains attached to a relatively small core protein [91]. Release of either free Hep chains or Hep-PG fragments from circulating cells into the blood or lymph networks would complement fixed tissue-based (SEC) monitoring of biomatrices and could indicate blood infections or wounding and the death of circulatory cells.

Unlike most of the other PGs residing within various tissues, DS-PGs are localized in the interstitial regions of almost all tissues examined [92]: the dermis, submucosal digestive tract layer, perichondral layer, perivascular connective tissue, perineurium, aortic adventitia, vein vessel walls, pleura, and the fibrous capsules of kidney and liver.

Thus, the three indicator GAG ligands may represent the circulatory systems and two types of biomatrix networks with many similar and some different components. The separate signaling inputs to the proposed stress sensing system from HA, DS and Hep may also be distinct, so that together their collective signaling input likely provides a more holistic, integrated, and cumulative assessment of the body’s overall biomatrix integrity and health. Thus, an integrated system monitoring the turnover of DS, Hep and HA from different types of tissue biomatrices, in a complementary way, could assess the health status of different biomatrices in almost all tissues of the body in a continuous ongoing manner. If others are able to follow up on this central idea with further investigations, I anticipate that the systemic responses elicited (18.5) will be proportional to the nature, level and type of GAG turnover detected by the proposed Tissue Stress Sensor System.

### 19.5. Biomatrix Turnover during Normal Homeostasis Does Not Activate Signaling

Our studies have shown that HA•HARE interactions stimulate NF-κB-activated gene expression [60,62,65,80] and that HARE activation only occurs within a narrow size range of HA degradation products [80]. The size range of HA draining from tissues into the lymphatic system under conditions of normal homeostasis [12,13] is typically very broad and such HA preparations do not activate HARE-mediated signaling [80]. Not surprisingly, HAase activity is only detectable at low levels in normal healthy tissues or fluids [12,13,93]. Therefore, under normal physiological conditions for a healthy person, the size range of HA released from biomatrices is very broad as random cleavages occur within MDa HA strands on cell surfaces and in biomatrices [12,13].

The most active 50–250 kDa signaling size HA is a small fraction of the total overall fragment sizes present in a continuous distribution of normally degraded HA fragments. If native HA sizes range up to 10^7^ Da and only 2–3 random cleavages of a bound HA are needed to enable its partial or total release from a biomatrix, then the released HA fragments likely span a >10^3^ Da mass range (e.g., 10^3^–10^6^ kDa). An important aspect of the proposed HA size-dependent sensing ability of HARE-mediated clearance is that, due to the competition for binding and uptake by smaller (<40 kDa) and larger (>400 kDa) nonsignaling HA, a mixture of broad HA size ranges does not initiate ERK1/2 and downstream gene activation (Section 15.2).

### 19.6. The Size of Degraded HA Is a Key Indicator of Normal Versus Pathological Biomatrix Turnover

In contrast to normal homeostasis, pathogen infections that cause extensive HA breakdown (e.g., by secreting HAase) or tissue damage due to wounding that causes cell death and release of endogenous lysosomal HAase could rapidly elevate the levels of 40–400 kDa HA fragments in the lymphatic and vascular circulations. The rapid HARE-mediated endocytosis by tissue SECs, would then activate NF-κB-mediated intracellular signaling responses and cytokine release.

### 19.7. Most Normal or Pathological Physiological Processes Involve Cytokines

NF-κB mediated transcription controls the synthesis and secretion or many cytokines that are crucially involved in numerous biological processes, including tissue development, maintenance of tissue homeostasis and apoptotic cell death of normal and malignant cells [87]. NF-κB is expressed ubiquitously, although its actions are regulated in stimulus-specific and cell-type-specific manners, allowing for diversely different effects and outcomes [88].

For example, NF-κB signaling in epithelial cells is important for maintaining immune homeostasis in skin and intestine, key barrier tissues [89]. In vulnerable tissues at external environment interfaces, NF-κB signaling performs vital keep-self-safe functions by regulating cell survival, barrier integrity, and how epithelial cells respond to ongoing immunologic and microbial challenges. Apoptosis (programmed cell death) is a normal physiological process critical for organ development, tissue homeostasis, and eliminating defective or potentially dangerous cells (e.g., cancer cells). In many cell types, NF-κB regulates apoptotic transcription programs either to induce apoptosis or, often more commonly, to block apoptosis [90].

## 20. Summary

Stab2 and HARE are just two of 28 recognized scavenger receptors [1], almost all of which are being studied to understand their roles in protecting and maintaining our health and physiologic homeostasis. As a group, SRs generally evoke less interest and funding devoted to understanding their function because they may appear to be secondary and less relevant to human health and longevity than the many significant causes of morbidity and mortality. We all understand at visceral levels based on personal and family experience the severity and toll of well recognized pathogenic diseases, cancers, heart disease, diabetes, etc. This list is long and funding priorities are understandably difficult to prioritize.

Significant unknowns, however, may be yet to be understood connections among some of the daily ongoing house-keeping functions maintained by the very complex SR networks and the very diseases and disorders at the forefront of our attention. We know our genetic blueprints, at the DNA level, for how we develop, survive and function are imperfect, but we also understand more and more how our thousands of controlling pathways and networks have evolved to become well integrated and connected. It would not be surprising if, in 20–30 years, many of our house-keeping functions are established as important or even vital in delaying or preventing some causes of morbidity and mortality.

## Figures and Tables

**Table 1:** HA or HEP endocytosis utilizes multiple HARE CD motifs. Details of the two studies are in Pandey et al. [58,59]. The organization and sequences of the four CD motifs targeting HARE to clathrin-coated pits (*M1–M4*) are shown in Figure 5. Results in boldface (Δ*M2* and Δ*M4*) indicate a motif that mediates endocytosis of either one of, but not both, HA and Hep.

HARE VARIANT	HA UPTAKE (%)	HEP UPTAKE (%)
WT	100	100
EV	0.2	0
Δ*M1*	51	65
Δ*M2*	**61**	**135**
Δ*M3*	44	35
Δ*M4*	**119**	**68**
Δ*M1M2*	61	-
Δ*M1M2M4*	58	-
Δ*M1M2M3M4*	7	-
WT(Y2519A)	94	95
Δ*M1M2M3* + Y2519A	5	0
+*M2* or +*M4*	0	0-5
+*M3*	58	65
+*M3*(Y2519A)	5	0

## Abbreviations

CD	cytoplasmic domain
CDR	chondroitin
CS	chondroitin sulfate
DS	dermatan sulfate
DxS	dextran sulfate
E1–E4	EGF-like repeat domains 1–4
EGF	Epidermal growth factor
ERK	Extracelluar Regulated Kinase
EV	empty vector
F1–F7	Fasciclin-1 domains 1–7
GAG	glycosaminoglycan
HA	Hyaluronan, hyaluronic acid
HAase	hyaluronidase
HARE	Hyaluronic Acid Receptor for Endocytosis (the half-length 190 kDa Stab2 receptor)
Hep	heparin
JNK	c-Jun N-terminal protein kinase
LYVE-1	lymphatic vessel endothelial cell HA receptor-1
LUC	luciferase
MS	mass spectrometry
NF-κB	Nuclear Factor-kappaB
PS	phosphatidyserine
SR	scavenger receptor
SEC	sinusoidal endothelial cell
Stab2	Stabilin-2
TMD	transmembrane domain
WT	wildtype
